# *Slc44a2* Deficiency Unveils an IFN‐I–Dependent Feedback Control of pDC Egress

**DOI:** 10.1002/advs.76325

**Published:** 2026-06-26

**Authors:** Ruiqun Chen, Tao Wu, Zhen Shi, Ravi Kumar Verma, Linlin Sheng, Lei Tao, Mingming Yang, Yuchen Wang, Conggang Zhang, Hao Fan, Li Wu, Ligong Chen

**Affiliations:** ^1^ State Key Laboratory of Membrane Biology School of Pharmaceutical Sciences Key Laboratory of Bioorganic Phosphorus Chemistry and Chemical Biology (Ministry of Education) Tsinghua Medicine Tsinghua University Beijing China; ^2^ Institute for Immunology School of Basic Medical Sciences Tsinghua Medicine Tsinghua University Beijing China; ^3^ Tsinghua‐Peking Center for Life Science Beijing China; ^4^ Department of Central Laboratory The First Hospital of Tsinghua University School of Clinical Medicine Tsinghua Medicine Tsinghua University Beijing China; ^5^ Bioinformatics Institute (BII) Agency for Science Technology and Research (A*STAR) Singapore; ^6^ School of Pharmaceutical Sciences Tsinghua Medicine Tsinghua University Beijing China; ^7^ Beijing Key Laboratory for Immunological Research on Chronic Diseases Beijing China; ^8^ State Key Laboratory of Metabolic Dysregulation & Prevention and Treatment of Esophageal Cancer Innovation Center of Basic Research for Metabolic‐Associated Fatty Liver Disease Ministry of Education of China Tianjian Laboratory of Advanced Biomedical Sciences Academy of Medical sciences Zhengzhou University Zhengzhou Henan China

**Keywords:** IFN‐I, negative feedback, pDC, pDC egress, SLC44A2

## Abstract

Plasmacytoid dendritic cells (pDCs) are a specialized subset of innate immune cells capable of sensing viral nucleic acids and rapidly producing large amounts of type I interferons (IFN‐I). However, excessive IFN‐I production can cause various immunopathogenic conditions. The capacity for IFN‐I production by pDCs is tightly regulated, yet the underlying mechanisms remain incompletely understood. Here, we describe two levels of negative regulatory mechanisms controlling IFN‐I production by pDCs. First, we identified SLC44A2 as a negative regulator of IFN‐I production. *Slc44a2* was highly expressed in resting pDCs but significantly downregulated upon activation. Deficiency of *Slc44a2* led to excessive IFN‐I production. Mechanistically, SLC44A2 may restrict IFN‐I production by exporting threonine, asparagine, and glutamine, amino acids that we found to be essential for IFN‐I production in pDCs. Second, we uncovered an IFN‐I‐dependent negative feedback mechanism controlling pDC egress. Excessive IFN‐I restrained pDC migration by downregulating CCR2 and CCR5. This feedback was generally observed during viral infections, autoimmune diseases, and in *Slc44a2*‐deficient mice. Taken together, these two regulatory mechanisms are essential for maintaining pDC homeostasis and preventing systemic overactivation of IFN‐I responses.

## Graphical abstract

Working model of SLC44A2‐mediated maintenance of pDC homeostasis. This model illustrates two central mechanisms by which SLC44A2 regulates pDC homeostasis: (1) SLC44A2 limits IFN‐I production by exporting amino acids (T, N, Q), thereby preventing spontaneous pDC activation. (2) Environmental IFN‐I sensing controls pDC migration: Elevated IFN‐I levels downregulate CCR2 and CCR5 expression, impairing pDC egress from the bone marrow to peripheral tissues. Together, these pathways prevent systemic IFN‐I hyperresponse and maintain pDC functional balance.

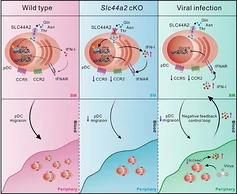

## 1 Introduction

Plasmacytoid dendritic cells (pDCs) represent a distinct subset of dendritic cells originating from hematopoietic stem cells in the bone marrow (BM), where they undergo full differentiation and maturation before migrating to peripheral tissues via the bloodstream [1]. Their primary role is to recognize viral or pathogenic nucleic acids, such as single‐stranded RNA or double‐stranded DNA, through Toll‐like receptors (TLR7/9) on endosomes, leading to rapid production of large amounts of type I interferons (IFN‐I) [2]. This capability is supported by their constitutive expression of high levels of IRF7, a key transcription factor regulating IFN‐I production in pDCs [3].

pDCs play pivotal roles in both antiviral innate immunity [4, 5]. During acute viral infections, such as lymphocytic choriomeningitis virus (LCMV), pDCs rapidly produce large quantities of IFN‐I [4]. In *Runx2* knockout mice, both pDC function and migration are significantly reduced, resulting in a significant decrease in IFN‐α in serum 24 h after LCMV virus infection, and an inability to effectively clear the virus [6]. However, excessive IFN‐I production can cause immunopathogenic conditions, such as systemic lupus erythematosus (SLE) and psoriasis, where pDCs can be activated by self‐DNA/RNA, and the excessive IFN‐I production exacerbates the progression of disease [7, 8, 9, 10]. In addition, during LCMV infection, the continuous secretion of IFN‐I by pDCs with preserved expression of LDHB can lead to pathological colitis [11]. Thus, pDC activation and IFN‐I secretion should be tightly regulated. Nowadays, several mechanisms have been identified for preventing systemic hyperresponse of IFN‐I. For instance, during viral infection, elevated IFN‐I can induce apoptosis of peripheral pDCs on the one hand [12], and restrict their development in the BM on the other hand [13], suggesting a feedback mechanism that controls IFN‐I levels. Despite these insights, the mechanisms underlying the negative regulation of pDC function in physiological and pathological contexts remain incompletely understood.

Solute carrier transporters (SLC) play crucial roles in immunity via modifying the metabolism and effector functions of immune cells [14, 15]. SLC44A2 belongs to the SLC44 family of choline transporters [16, 17, 18], and has been implicated in several diseases and biological process, including Meniere's disease [18, 19, 20, 21], transfusion‐related acute lung injury [22, 23], venous thromboembolism [24, 25], hemostasis [26], platelet activation [27], neutrophil extracellular trap formation (NETosis) [28, 29], and phenotypic switching of vascular smooth muscle cells (VSMCs) [30].

Here, we found that *Slc44a2* is highly expressed in pDCs, but significantly decreased upon activation. Deletion of *Slc44a2* led to activation of the IFN‐I signaling pathway in pDCs even under resting conditions, and demonstrated an enhanced ability to produce IFN‐I upon activation. Moreover, *Slc44a2*‐deficient pDCs showed higher intracellular levels of threonine (T), asparagine (N), and glutamine (Q), amino acids we found that were required for IFN‐I production. Thus, we suggest that SLC44A2 functions as a negative regulator of IFN‐I production, possibly by exporting amino acids required for IFN‐I production. Additionally, *Slc44a2*‐deficient mice showed lower pDC numbers in peripheral tissues. We further confirmed that enhanced IFN‐I signaling in *Slc44a2*‐deficient pDCs restrained their egress via downregulation of CCR2 and CCR5. Consistently, decreased CCR2 and CCR5 expression on BM pDCs was also observed during viral infection and autoimmune diseases, accompanied by a marked reduction in peripheral pDCs. Therefore, we suggest that BM pDCs can sense environmental IFN‐I levels and adjust their egress accordingly. This may represent a vital feedback mechanism for maintaining pDC homeostasis and preventing excessive IFN‐I production.

## 2 Results

### 2.1 *Slc44a2* Deficiency Reduces pDC Numbers in the Peripheral Tissues

The SLC family, the largest and most substrate‐diverse superfamily of transporters, has recently been shown to influence the function and development of dendritic cells (DCs) [14, 21, 31, 32, 33]. Considering the role of metabolic regulation in these processes [34, 35], we screened for genes associated with metabolic pathways and the SLC family (Figure S1A). This analysis identified five SLC genes shared between mouse and human pDCs (Figure S1B,C). Among these, *Slc44a2* exhibited the highest expression, a finding corroborated using the ImmGen database (Figure 1A and Figure S1D). Furthermore, RT‐qPCR analysis confirmed that  Slc44a2 expression was significantly higher in pDCs than in cDCs (Figure 1B). Furthermore, we analyzed our in‐house proteomic dataset of mouse splenic DC subsets, which revealed that SLC44A2 protein abundance was also highest in pDCs (Figure 1C). Phylogenetic analysis further revealed that SLC44A2 orthologs were also highly conserved across jawed vertebrates with functional type I interferon systems, including fish, reptiles, birds, and bats (Figure S2) [36, 37, 38], suggesting that the regulatory role of SLC44A2 in pDC biology is ancient. Notably, we also found a significant decrease in *Slc44a2* expression in splenic pDCs after viral infection (Figure 1D). Despite these findings, the regulatory role of SLC44A2 in the development or function of pDCs during homeostasis and viral infection is not well understood.

**FIGURE 1:**
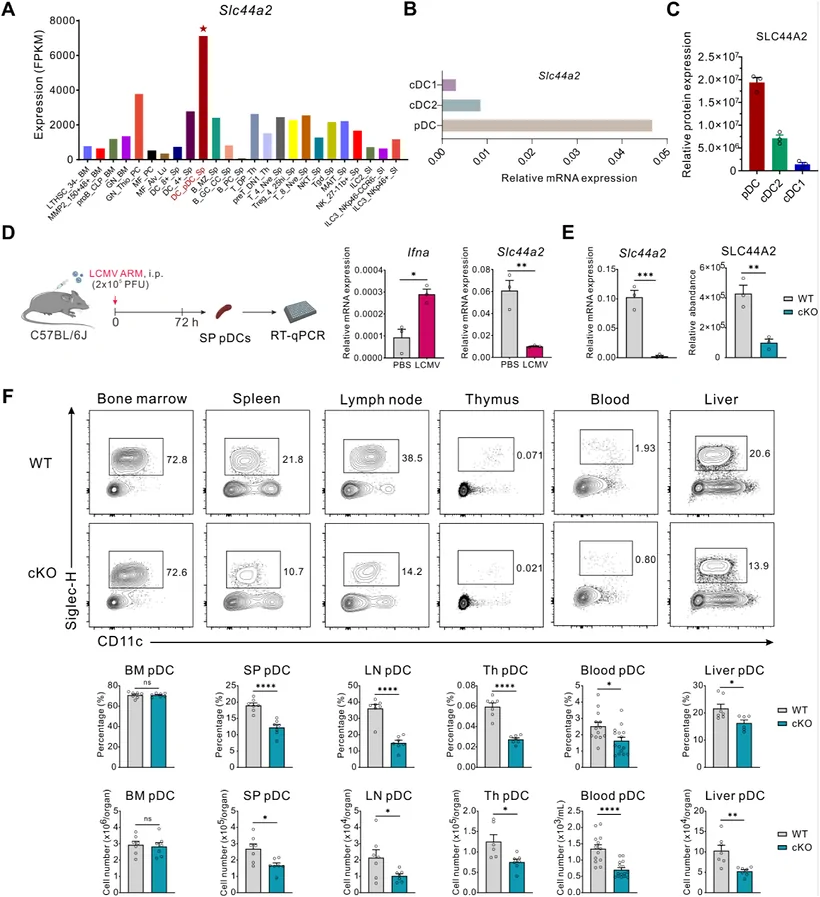
*Slc44a2* deletion reduces pDC numbers in the peripheral tissues. (A) *Slc44a2* expression among the major hematopoietic progenitor cells and immune cells. Data from ImmGen database. (B) Expression of *Slc44a2* in mouse splenic DC subsets assessed by RT‐qPCR analysis. (C) Expression of SLC44A2 in mouse splenic DC subsets assessed by DIA‐based proteomic analysis (n = 3). (D) The changes in *Ifna* and *Slc44a2* in splenic pDCs were detected by RT‐qPCR after 72 h of infection with LCMV‐Arm virus (2 × 10^5^ PFU/mouse, n = 3). (E) The knockout efficiency of *Slc44a2* in pDCs from *Slc44a2‐*deficient mice was evaluated by RT‐qPCR and DIA‐based proteomic analysis (n = 3). (F) The representative flow plots, proportions, and cell numbers of pDCs in the bone marrow (BM), spleen (SP), inguinal lymph nodes (LN), thymus (Th), blood, and liver of WT and cKO mice (BM, SP, LN, Th, Liver, n = 6–7; Blood, n = 13–15). pDCs were defined by live (7‐AAD^−^) CD45^+^ CD11b^−^ CD3e^−^ CD19^−^ CD11c^int^ Siglec‐H^+^ cells. The percentage of pDCs in the BM, SP, LN, and Th was calculated as the proportion of CD11c^int^ Siglec‐H^+^ cells among live (7‐AAD^−^) CD45^+^ CD11b^−^ CD3e^−^ CD19^−^ cells. Graphs represented mean ± SEM, with individual data points shown for each mouse. *p* values were determined by unpaired two‐tailed Student's *t*‐test of n = 3 independent biological experiments. ^*^ *p* < 0.05, ^**^ *p* < 0.01, ^***^ *p* < 0.001, ^****^ *p* < 0.0001; ns, not significant.

To investigate the potential role of *Slc44a2* in pDCs, we generated mice with hematopoietic system‐specific knockout of *Slc44a2* (*Slc44a2* ^fl/fl^ *Vav* ^iCre^). For simplicity, these mice will be referred to as cKO, and their littermate controls (*Slc44a2* ^fl/fl^) as WT. The knockout efficiency of *Slc44a2* exceeded 98% (Figure 1E). In cKO mice, the proportion and number of BM pDCs (defined as CD11c^int^ Siglec‐H^+^ pDCs) remained unchanged, while significant reductions were observed in the spleen (SP), inguinal lymph nodes (LN), thymus (Th), the blood, and the liver (Figure 1F). Flow cytometric analysis using another specific marker for pDCs, PDCA‐1 (gating on CD11c^int^ PDCA‐1^+^ pDCs), also corroborated these findings (Figure S3A). Consistently, immunofluorescence analysis also showed no significant change in the number of BM pDCs, but a marked reduction of pDCs in the spleen, inguinal lymph nodes, and thymus (Figure S3B). These findings indicated that while *Slc44a2* knockout did not affect the number of pDCs in the BM, it led to a significant reduction of pDCs in the peripheral tissues.

Although *Vav‐iCre* mediated the deletion of *Slc44a2* across the entire hematopoietic system, we observed no significant changes in other major immune cell types, except for a slight increase in NK cells (Figure S4A,B). To further confirm the effect of *Slc44a2* knockout on pDCs, we also analyzed the phenotype of pDCs in the *Slc44a2* ^fl/fl^ *CD11c* ^Cre^ mice, in which *Slc44a2* was selectively knocked out in cDCs and pDCs (Figure S5A). Similar to the cKO mice, these mice exhibited a significant reduction in peripheral pDCs but no changes in BM pDCs (Figure S5B,C). Other immune cells, including cDCs, macrophages, NK cells, B cells, and T cells, showed no significant changes in the *Slc44a2* ^fl/fl^ *CD11c* ^Cre^ mice as well (Figure S5D). These findings collectively demonstrated that SLC44A2 regulates the homeostasis of peripheral pDCs.

The above results suggest that *Slc44a2* deficiency does not significantly affect the development of BM‐derived pDCs. Consistent with this, *Slc44a2* deletion did not significantly impact the development of pDCs or cDCs during in vitro Flt3L‐induced DC differentiation (Figure S6A–C). We next comprehensively assessed apoptosis and proliferation of pDCs in the BM. The frequency of Ki67^+^ pDCs in the BM was comparable between WT and cKO mice, indicating no defect in proliferation (Figure S7A). Similarly, the proportions of early apoptotic (Annexin V^+^PI^−^) and late apoptotic (Annexin V^+^PI^+^) BM pDCs were unchanged in cKO mice (Figure S7B), and FL‐pDCs differentiated in vitro showed no difference in apoptosis (Figure S7C). Together, these findings indicate that *Slc44a2* is dispensable for the development, proliferation, and survival of pDCs in the BM.

To extend this analysis to peripheral organs, we examined pDC apoptosis in the spleen, inguinal lymph nodes, and thymus. Notably, splenic pDCs from cKO mice displayed significantly increased apoptosis, whereas no changes were detected in the inguinal lymph nodes or thymus (Figure S7B). Although enhanced splenic apoptosis may partially contribute to the reduced splenic pDC pool, pDC numbers were decreased across all peripheral organs examined. Given that apoptosis was selectively increased only in the spleen, while BM proliferation and survival remained intact, these findings support defective BM egress as the primary mechanism underlying the systemic reduction of peripheral pDCs in cKO mice. Collectively, our data suggest that *Slc44a2* deficiency disrupts pDC homeostasis predominantly through impaired BM egress, with an additional contribution from enhanced splenic apoptosis.

### 2.2 *Slc44a2* Deficiency Restricts pDC Egress Through Downregulation of *Ccr2* and *Ccr5*

To further investigate the effects of *Slc44a2* knockout on pDCs, we sorted BM pDCs from WT and cKO mice and performed RNA‐seq analysis (Figure S8A). The results revealed significant upregulation of *Ly6a* (i.e., *Sca‐1*), *Cd69*, *Icam1*, *Kdr*, *Cd40*, and *Cd86* in the cKO group (Figure S8B), which are associated with pDC maturation or activation [39, 40]. The changes in these markers were further confirmed by flow cytometric analysis (Figure 2A and Figure S8C,D). Since Sca‐1 is a marker of mature pDCs that are mainly distributed in the peripheral tissues, while Sca‐1^−^ pDCs are predominantly located in the BM [41], we used Sca‐1 expression level to evaluate the maturation status of BM pDCs. A significantly higher proportion and number of Sca‐1^+^ pDCs were found in the BM of cKO mice (Figure 2B and Figure S8C). The levels of Sca‐1 expression on pDCs in the spleen, inguinal lymph nodes, and thymus remained consistently high and showed no significant changes upon *Slc44a2* deletion (Figure 2C). This retention of Sca‐1^+^ pDCs in the BM corresponded to their reduction in peripheral tissues (Figure 2D). Hence, the findings suggested that the higher number of mature Sca‐1^+^ pDCs in the BM and the reduced number in peripheral tissues may result from the retention of Sca‐1^+^ pDCs in the BM of *Slc44a2* knockout mice.

**FIGURE 2:**
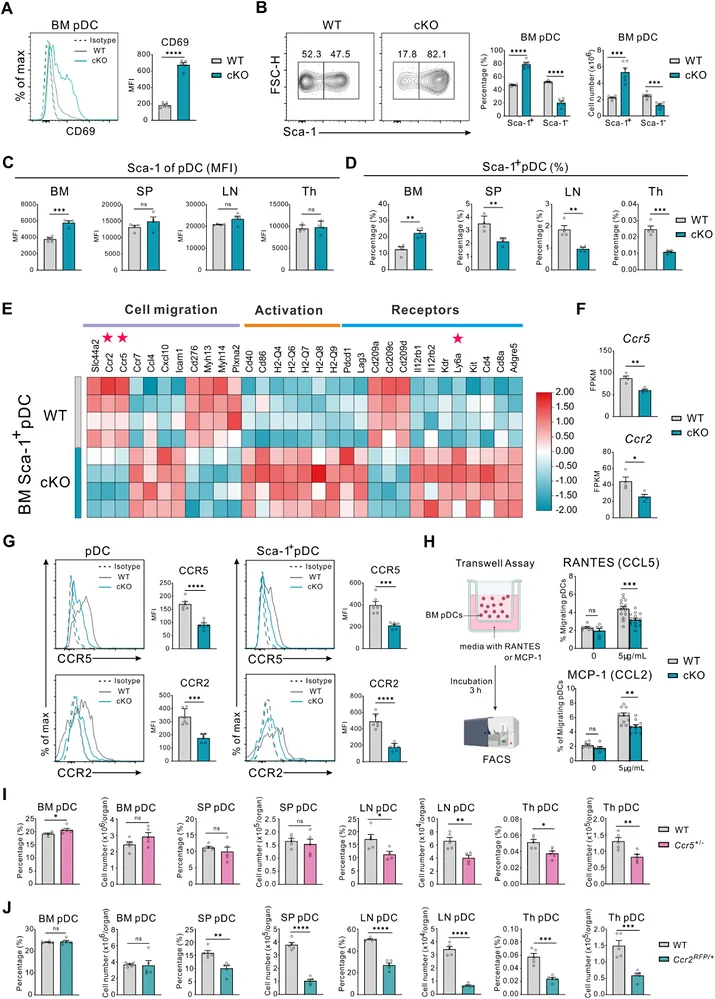
*Slc44a2* deficiency impedes pDC egress from the BM via downregulation of *Ccr2* and *Ccr5*. (A) Mean fluorescence intensity (MFI) of CD69 on BM pDCs of WT and cKO mice (n = 5). (B) The flow plots, proportions, and cell numbers of Sca‐1^+^ and Sca‐1^−^ pDCs in the BM of WT and cKO mice (n = 5–6). Frequency of Sca‐1^+^ pDCs in BM, calculated as the percentage of live (7‐AAD^−^) CD45^+^ CD11b^−^ CD3e^−^ CD19^−^ CD11c^int^ Siglec‐H^+^ cells. (C) MFI of Sca‐1 on pDCs isolated from the BM, SP, LN, and Th of WT and cKO mice (n = 4). (D) The proportions of Sca‐1^+^ pDCs in the BM, SP, LN, and Th from WT and cKO mice (n = 4). The percentage of Sca‐1^+^ pDCs in different tissues was calculated as the proportion of Sca‐1^+^ pDCs among live (7‐AAD^−^) CD45^+^ CD11b^−^ CD3e^−^ CD19^−^ cells. (E) Heatmap showing the selected differentially expressed genes associated with the migration, activation, or receptors of Sca‐1^+^ pDCs in the BM from WT and cKO mice (n = 4). (F) Expression of *Ccr5* and *Ccr2* in BM Sca‐1^+^ pDCs, FPKM values derived from the RNA‐seq data (n = 4). (G) MFI of CCR5 and CCR2 on Sca‐1^+^ pDCs in the BM of WT and cKO mice (n = 6). (H) Transwell migration assays of BM pDCs in medium containing RANTES (a CCR5 ligand) and MCP‐1 (a CCR2 ligand) at a concentration of 5 µg/mL (n = 6–15). (I) Proportions and absolute numbers of pDCs in the BM, SP, LN, and Th of *Ccr5* ^+/−^ heterozygous mice (n = 5). pDCs were defined by live (7‐AAD^−^) CD45^+^ CD11b^−^CD3e^−^CD19^−^CD11c^int^ Siglec‐H^+^ cells. (J) Frequencies and cell counts of pDCs in the BM, SP, LN, and Th of *Ccr2^RFP/+^* heterozygous mice (n = 5). pDCs were defined by live (7‐AAD^−^) CD45^+^ CD11b^−^CD3e^−^CD19^−^CD11c^int^ Siglec‐H^+^ cells. Data were expressed as mean ± SEM. Symbols represented individual mice. Statistical significance was determined by unpaired two‐tailed Student's *t*‐test of n = 3 independent biological experiments. ^*^ *p* < 0.05, ^**^ *p* < 0.01, ^***^ *p* < 0.001, ^****^ *p* < 0.0001; ns, not significant.

To determine the cause of retention of Sca‐1^+^ pDCs in the BM of *Slc44a2* knockout mice, we used RNA‐seq analysis to compare the alterations in genes after *Slc44a2* deletion (Figure 2E). The results revealed a marked downregulation of *Ccr5* and *Ccr2* expression in the BM Sca‐1^+^ pDCs of cKO mice (Figure 2F). Flow cytometric analysis also confirmed that *Slc44a2* deficiency led to a significant reduction in CCR5 and CCR2 expression on pDCs (Figure 2G). Consistently, transwell migration assays further demonstrated that pDCs from cKO mice exhibited significantly impaired migration in response to RANTES (a CCR5 ligand) and MCP‐1 (a CCR2 ligand) (Figure 2H). CCR5 and CCR2 were shown to mediate the migration of pDCs [39, 42]. Accordingly, mice treated in vivo with cenicriviroc (CVC), a dual CCR2/CCR5 antagonist [42, 43, 44, 45], showed a significantly reduced proportion of pDCs in the spleen, inguinal lymph nodes, and thymus (Figure S9A). However, it did not significantly affect the proportion of pDCs in the BM (Figure S9A). Thus, inhibiting CCR2 and CCR5 impeded the migration of pDCs from the BM to the periphery.

Since *Ccr5* and *Ccr2* were encoded by adjacent homologous genes with 71% sequence similarity, functional redundancy may exist [43]. To further assess their roles in pDC migration, we used *Ccr5* knockout (*Ccr5* ^−/−^) and *Ccr2* knockout (*Ccr2* ^RFP/RFP^) mice. Both knockout mice exhibited a significant reduction in the proportion and number of pDCs in the spleen, inguinal lymph nodes, and thymus, but no significant changes in BM pDCs (Figure S9B,C). Notably, *Ccr5* ^+/−^ heterozygous mice showed ∼50% reduction in CCR5 levels (Figure S9D,E), and the *Ccr5* knockout had no effect on *Ccr2* expression (Figure S9F). *Ccr2* ^RFP/+^ heterozygous mice showed significantly reduced pDC proportions and numbers in the spleen, inguinal lymph nodes, and thymus, while *Ccr5* ^+/−^ heterozygous mice only showed remarkably reduced pDC proportions and numbers in inguinal lymph nodes and thymus, with a more pronounced effect in *Ccr2* ^RFP/+^ heterozygous mice (Figure 2I,J). This evidence indicated that CCR2 played a more critical role than CCR5 in pDC migration to the peripheral tissues. Given that *Slc44a2* deficiency reduced CCR5 and CCR2 levels by ∼50% in BM pDCs, we generated *Ccr5* ^+/−^ *Ccr2* ^RFP/+^ double‐heterozygous mice to mimic the phenotype of cKO mice. These mice exhibited peripheral pDC reduction similar to that observed in *Slc44a2* knockout mice (Figure S9G). Our results therefore suggested that decreased CCR5 and CCR2 expression on pDCs in *Slc44a2* knockout mice might account for the impaired pDC migration from the BM.

In addition to CCR2 and CCR5, several chemokine receptors can also regulate pDC egress from the BM [46, 47, 48, 49], such as CXCR4 [6, 50, 51], CXCR3 [52], CCR7 [53, 54, 55], and CCR9 [56]. Among those, CXCR4, which is suppressed by the transcription factor RUNX2, is one key factor for retaining pDCs in the BM [6, 50, 51]. However, in *Slc44a2*‐deficient mice, the expression of RUNX2 and CXCR4 by BM pDCs remained unchanged (Figure S10A–C), and migration mediated by the CXCR4‐CXCL12 axis remained unaffected (Figure S10D). While *Slc44a2*‐deficient pDCs exhibited decreased CXCR3 expression and increased CCR7 expression (Figure S10E,G), their response to CXCL9 (a CXCR3 ligand) and CCL21 (a CCR7 ligand) was comparable to that of WT pDCs (Figure S10F,H). Furthermore, F‐actin polarization and the migratory assays following CCL21 stimulation revealed no significant differences between WT and cKO pDCs, indicating comparable motility (Figure S10I,J). Moreover, we observed changes in adhesion molecules and integrins [57, 58], including increased ICAM‐1 (Figure S10K) and decreased Integrin β2 (CD18) (Figure S10L). However, no significant changes were observed in the levels of integrin αX (CD11c), integrin αL (CD11a), and integrin α4 (CD49d) (Figure S10L). Further studies are warranted to determine whether these molecules are involved in pDC egress from the BM.

In summary, *Slc44a2* knockout did not affect pDC motility but reduced CCR2 and CCR5 expression on BM pDCs, hindering their migration from the BM to peripheral tissues and leading to decreased numbers of pDCs in several peripheral tissues.

### 2.3 *Slc44a2* Deletion Enhances Type I Interferon Signaling in pDCs

To further explore how SLC44A2 regulated CCR2 and CCR5, we re‐analyzed the differentially expressed genes in BM pDCs from cKO mice (Figure S11A). This revealed a significant upregulation of *Irf7*, a key regulator of IFN‐I production in pDCs [3]. (Figure S11B). Flow cytometry analysis also confirmed increased IRF7 expression in cKO BM pDCs (Figure S11C), suggesting enhanced type I IFN secretion capacity of BM pDCs. We also observed elevated expression of other IFN‐I‐related genes, including *Stat1*, *Stat2*, *Isg15*, and *Isg20* in cKO BM pDCs (Figure S11D). Since *Slc44a2* deletion increased the proportion of mature Sca‐1^+^ pDCs in the BM, the upregulation of these genes could be attributed to the higher number of mature pDCs in cKO mice.

To further evaluate the impact of *Slc44a2* knockout on pDC function, we analyzed the RNA‐seq data of BM Sca‐1^+^ pDCs from WT and cKO mice. *Slc44a2* deletion also significantly increased the RNA levels of *Irf7*, *Stat1*, *Stat2*, *Zbp1*, and multiple ISGs in the BM Sca‐1^+^ pDCs (Figure 3A,B). KEGG pathway enrichment analysis revealed 15 upregulated pathways, including the JAK‐STAT signaling pathway, RIG‐I‐like receptor signaling pathway, and antiviral‐related pathways, etc (Figure 3C). Gene Set Enrichment Analysis (GSEA) indicated an enhanced activation of type I interferon‐related pathways in cKO pDCs (Figure 3D). Again, *Ifna2* level was significantly elevated in Sca‐1^+^ pDCs of the cKO mice (Figure 3E). Notably, *Opn* (osteopontin), a molecule essential for TLR9‐dependent IFN‐α production in pDCs [59, 60], was also significantly upregulated in cKO Sca‐1^+^ pDCs (Figure S11E). However, the expression of other type I IFN subtypes (*Ifna1*, *Ifna4*, *Ifna5*, *Ifna6*, *Ifna9*, *Ifna11*, *Ifna14*, *Ifnab*, *Ifnk*, *Ifnz*) and type III IFNs (*Ifnl1*, *Ifnl2*, *Ifnl3*) [61] remained largely unchanged in cKO Sca‐1^+^ pDCs (Figure S11F). Moreover, when stimulated with CpG‐A, *Slc44a2‐*deficient Sca‐1^+^ pDCs showed enhanced IFN‐α secretion (Figure 3F). These data thus demonstrated that *Slc44a2* knockout enhances the production of type I interferon by pDCs.

**FIGURE 3:**
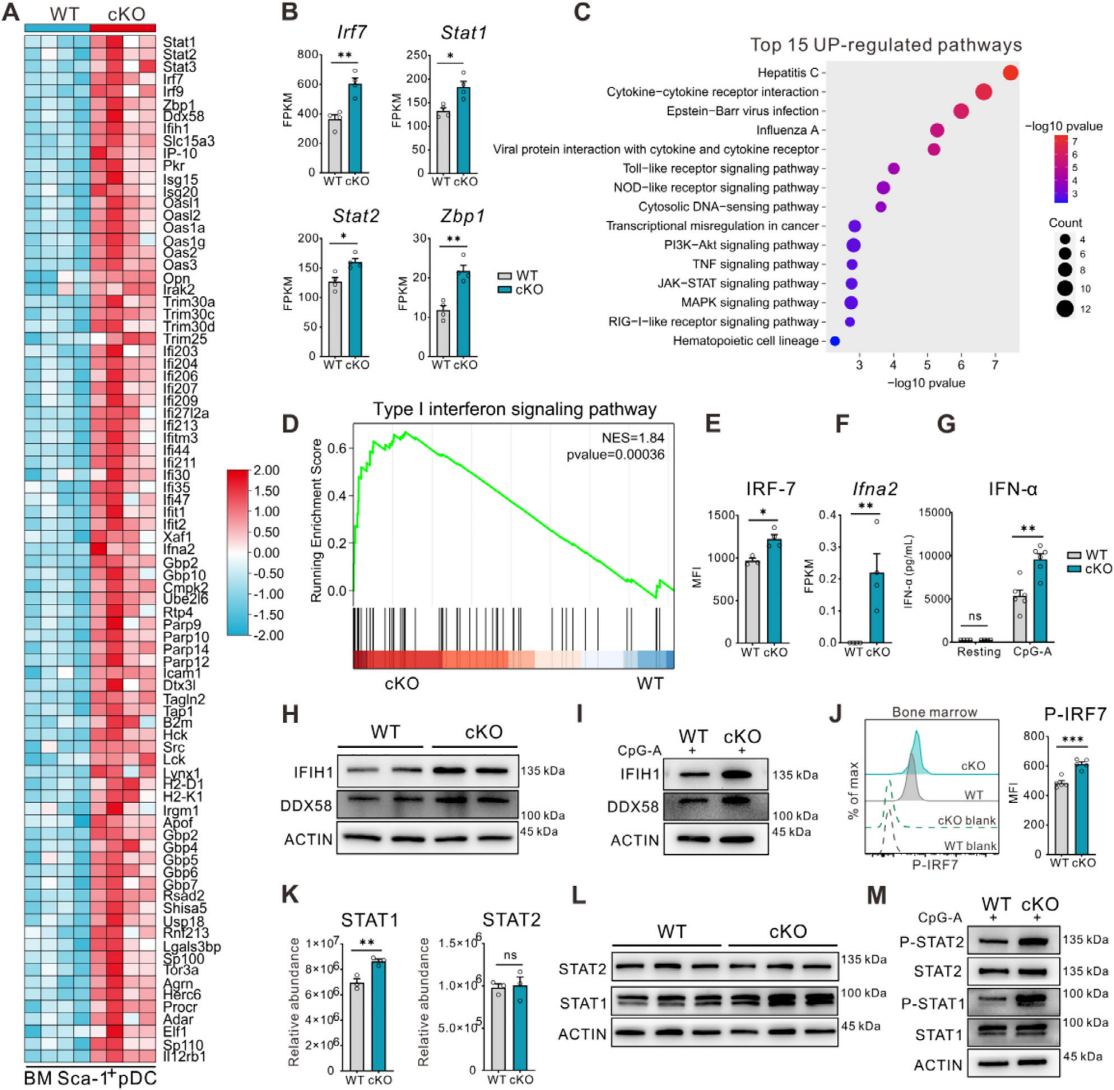
*Slc44a2* deficiency enhances type I interferon signaling in pDCs. (A) Heatmap illustrating differentially upregulated genes associated with pDC function in the RNA‐seq dataset of BM Sca‐1^+^ pDCs isolated from WT and cKO mice (n = 4). (B) Expression levels (FPKM value) of *Irf7, Stat1*, *Stat2*, and *Zbp1* in BM Sca‐1^+^ pDCs from WT and cKO mice (n = 4). (C) Pathway enrichment analysis of differentially upregulated genes in the RNA‐seq dataset from BM Sca‐1^+^ pDCs of WT and cKO mice, with the top 15 enriched pathways listed. (D) Enriched type I interferon signaling pathway in cKO pDCs revealed by Gene Set Enrichment Analysis (GSEA). NES: Normalized Enrichment Score. (E) MFI of IRF‐7 expression in BM Sca‐1^+^ pDCs from WT and cKO mice (n = 3–4). (F) Expression (FPKM value) of *Ifna2* and *Opn* in BM Sca‐1^+^ pDCs of WT and cKO mice (n = 4). (G) IFN‐α secretion by BM Sca‐1^+^ pDCs from WT and cKO mice was measured by ELISA under steady‐state conditions and following 24 h CpG‐A (1 µM) stimulation (n = 4–6). (H,I) Western blot analysis of IFIH1 and DDX58 in WT and cKO BM pDCs under the steady state (H) and following 2.5 h CpG‐A (1 µM) stimulation (I). (J) Intracellular staining and MFI of phosphorylated IRF7 (P‐IRF7) in BM pDCs from WT and cKO mice (n = 5). (K) Relative protein levels of STAT1 and STAT2 expression in BM pDCs from WT and cKO mice were analyzed using DIA‐based proteomics (n = 3). (L,M) Protein levels of STAT1, STAT2, P‐STAT1, and P‐STAT2 in BM pDCs from WT and cKO mice were analyzed by western blot under steady‐state conditions (L) and after 2.5 h CpG‐A (1 µM) stimulation (M). Data were expressed as mean ± SEM. Symbols represent individual mice. Statistical significance was determined by unpaired two‐tailed Student's *t*‐test of n = 3 independent biological experiments. ^*^ *p* < 0.05, ^**^ *p* < 0.01, ^***^ *p* < 0.001; ns, not significant.

Current understanding holds that pDCs primarily recognize nucleic acids via the highly expressed endosomal receptors TLR7 and TLR9, leading to their activation and IFN‐I secretion [62]. To assess whether TLR7/9 signaling contributes to pDC activation under steady‐state conditions, we analyzed RNA‐seq and proteomics data from BM pDCs of WT and cKO mice. We found that deletion of *Slc44a2* led to a significant upregulation of *Tlr9* and *Myd88* at the transcript level, whereas the expression of *Tlr7*, *Irak1*, *Irak4*, and *Traf6* remained unchanged (Figure S11G). However, none of these molecules exhibited significant changes at the protein level (Figure S11H). Furthermore, the activation/maturation markers (e.g., Sca‐1, KDR, ICAM‐1, MHC‐II, and CD40) were upregulated in resting cKO BM pDCs, whereas treatment with the MyD88 inhibitor ST2825 failed to diminish the elevated expression of these markers (Figure S11I). Therefore, these data indicated that pDC activation in *Slc44a2*‐deficient mice under steady‐state conditions occurs independently of the TLR7/9 pathway.

Type I interferon production in pDCs occurs through multiple steps. Key sensors such as DDX58, IFIH1, and IFI203‐IFI205 detect RNA and DNA in the cytoplasm to initiate the process [63, 64, 65]. Secreted IFN‐α further amplifies its production through an autocrine pathway by binding to its receptor (IFNAR), activating the JAK‐STAT pathway, and inducing the expression of ISGs such as DDX58, IFIH1, and IRF7 [66, 67]. In our study, *Slc44a2*‐deficient pDCs showed elevated protein levels of DDX58 and IFIH1, with the increase being more pronounced after CpG‐A stimulation (Figure S11J). Similarly, IRF7 and its phosphorylated form (P‐IRF7) were markedly elevated (Figure 3H and Figure S11K). Consistent with the enriched JAK‐STAT pathway in cKO pDCs predicted by the RNA‐seq analysis (Figure 3C), we observed increased STAT1 expression upon *Slc44a2* knockout, while no significant change in STAT2 levels by proteomics analysis (Figure S11L). That was further confirmed by western blot (Figure 3I). Notably, both P‐STAT1 and P‐STAT2 levels were significantly increased in *Slc44a2*‐deficient pDCs upon CpG‐A stimulation (Figure 3J). Additionally, the RNA levels of type I interferon receptors (*Ifnar1* and *Ifnar2*) and JAK family members (*Jak1*, *Jak2*, and *Jak3*) were not significantly changed in cKO pDCs (Figure S11M). These results suggested that *Slc44a2* knockout boosts IFN‐α secretion by activating the JAK‐STAT signaling pathway without affecting the expression of major signaling member proteins.

To further assess whether the enhanced IFN‐I production by *Slc44a2*‐deficient pDCs acts in a paracrine manner, we sorted non‐pDC immune cells from the bone marrow and spleen of WT and cKO mice at steady state. RT‐qPCR analysis revealed that a panel of representative ISGs, including *Isg15*, *Mx1*, *Cxcl10*, *Oas1a*, and *Ifit1*, were all significantly upregulated in the non‐pDC compartment of cKO mice compared with WT controls (Figure S12A). These data support the presence of paracrine IFN‐I signaling in *Slc44a2*‐deficient mice under basal conditions. In contrast, following CpG‐A challenge, ISG expression was robustly induced in both genotypes, with no significant difference observed between WT and cKO mice (Figure S12B), suggesting that acute stimulation overrides the modest differences seen at steady‐state.

Given the enhanced IFN‐I production capacity of *Slc44a2*‐deficient pDCs despite their reduced peripheral abundance, we next assessed the net systemic IFN‐α response in vivo. Following intravenous CpG‐A injection, serum IFN‐α levels were significantly lower in cKO mice compared with WT controls at both 6 and 12 h post‐injection (Figure S13A), indicating that pDC number is the dominant determinant of systemic IFN‐α output during acute pDC‐driven stimulation. To further evaluate the overall antiviral response, WT and cKO mice were intraperitoneally infected with the LCMV‐ARM virus, and serum IFN‐α levels and viral loads were measured at 12, 24, and 72 h. In contrast to the CpG‐A model, no significant differences were observed in serum IFN‐α levels at 12, 24, and 72 h post‐infection (Figure S13B), and viral loads in the liver and spleen were comparable between genotypes at all time points examined (Figure S13C). Of note, at 24 h post‐LCMV infection, cKO mice exhibited a non‐significant trend toward lower serum IFN‐α, which likely reflects the residual influence of reduced peripheral pDC numbers in the early phase of infection. Consistent with the late‐phase LCMV results, *Slc44a2* deficiency had no impact on the development of SLE (Figure S13D). Together, these data suggest that reduced peripheral pDC abundance limits systemic IFN‐I production during acute pDC‐centric responses, whereas during prolonged viral infection or chronic inflammatory conditions, compensatory IFN‐I production is sufficient to maintain overall systemic IFN‐α levels.

### 2.4 Activation of Type I IFN Signaling Restrains pDC Egress Through Downregulation of CCR2 and CCR5

To further explore the potential relationship between the enhanced IFN‐I production and the reduced migration of pDCs in *Slc44a2*‐deficient mice, we first examined the dynamic expression of *Ifna*, *Ccr2*, *Ccr5*, and *Slc44a2* in BM pDCs after CpG‐A stimulation. Consistent with the published studies [34], the level of *Ifna* mRNA was initially increased and then decreased in pDCs following CpG‐A stimulation (Figure S14A). Correspondingly, the level of IFN‐α in the supernatant continuously accumulated with the extension of stimulation time (Figure S14A). Meanwhile, we observed a continuous decline of *Ccr2* and *Ccr5* expression (Figure S14B), indicating their dramatic suppression in pDCs activated by CpG‐A, whereas *Slc44a2* mRNA levels showed an initial decrease followed by partial recovery (Figure S14B). To confirm whether IFN‐I accounts for the suppression of *Ccr2* and *Ccr5* during pDC activation, we next treated BM pDCs with IFN‐α directly and observed a significantly decreased expression of both *Ccr2* and *Ccr5* (Figure 4A,B). However, *Slc44a2* expression was not significantly affected by IFN‐α (Figure 4B). Furthermore, we used an IFNAR1 neutralizing antibody to block IFN‐I signaling and observed an efficient impairment of IFN‐I production by activated pDCs (Figure S14C). Consistently, blocking IFNAR1 restored *Ccr2* and *Ccr5* mRNA expression in activated pDCs (Figure S14D), while showing no obvious effect on the expression of *Slc44a2* (Figure S14D). Moreover, when wild‐type mice were injected with IFN‐α, a significant reduction in CCR2 and CCR5 expression on BM pDCs was also observed, accompanied by a significant increase in the proportion of BM Sca‐1^+^ pDCs (Figure 4C–E). These findings demonstrated that IFN‐I produced by activated pDCs suppresses the expression of *Ccr2* and *Ccr5* in pDCs.

**FIGURE 4:**
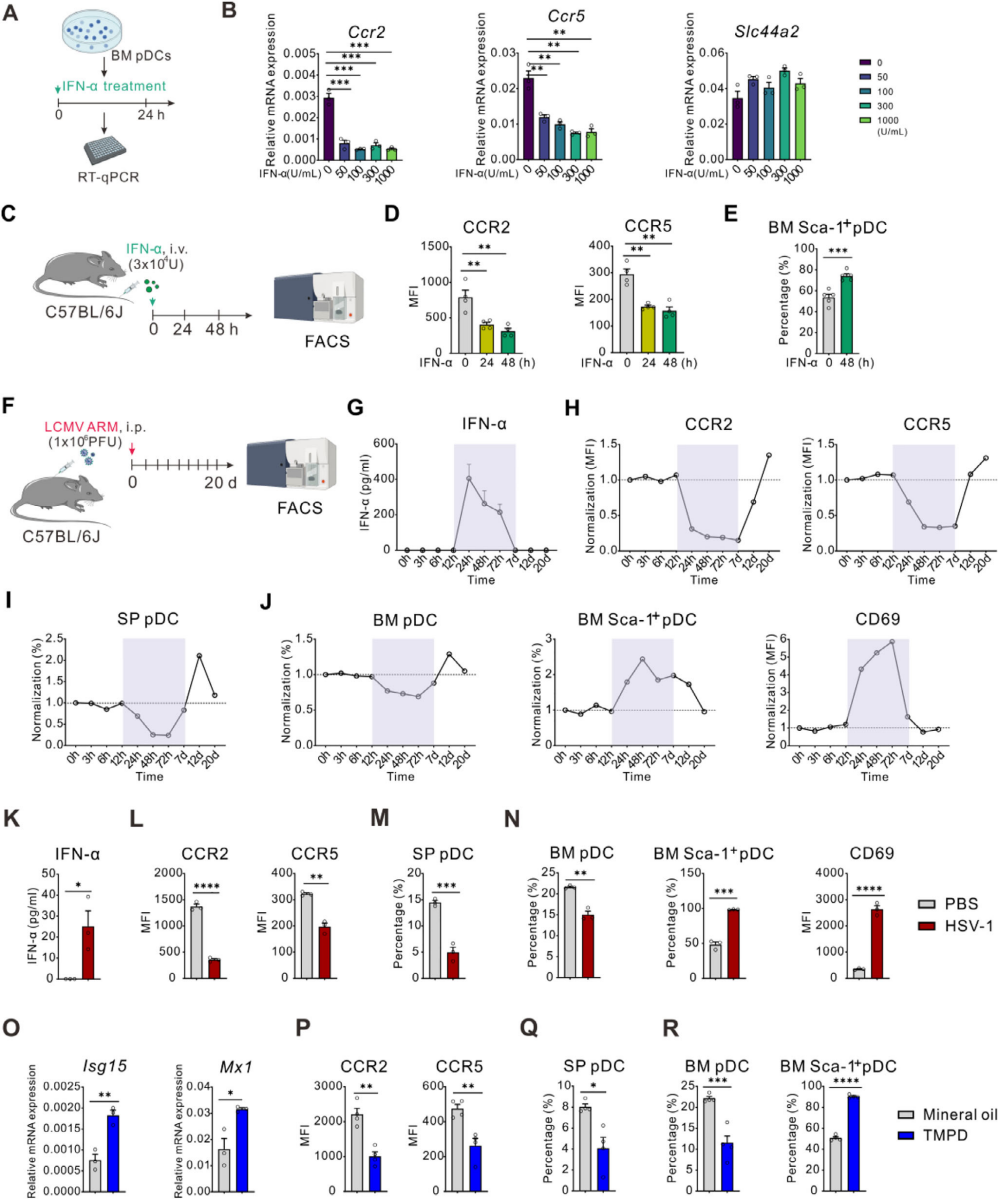
Activation of type I IFN signaling restrains pDC egress via downregulation of CCR2 and CCR5. (A,B) Expression of *Ccr2*, *Ccr5*, and *Slc44a2* in BM pDCs treated with various concentrations of IFN‐α for 24 h (n = 3). (C,D) CCR2 and CCR5 expression on BM pDCs from WT mice at 24 and 48 h after being intravenously injected (i.v.) with IFN‐α (3 × 10^4^ U/mouse, n = 4). (E) Percentages of BM Sca‐1^+^ pDCs from WT mice at 48 h after being intravenously injected (i.v.) with IFN‐α (3 × 10^4^ U/mouse, n = 5). (F) Experimental schematic diagram of LCMV infection: mice were intraperitoneally injected (i.p.) with LCMV virus (1 × 10^6^ PFU/mouse) for various times (n = 3). (G) Serum IFN‐α levels in C57BL/6J mice at indicated times post PBS injection or LCMV infection. (H) Expression levels of CCR2 and CCR5 on BM pDCs at indicated times post‐infection. (I) Proportion of splenic pDCs at indicated times post‐LCMV infection. (J) Percentage of BM pDCs and BM Sca‐1^+^ pDCs, and CD69 expression at indicated times post‐LCMV infection. Values (H–J) represent the ratios of the LCMV‐infected group normalized to PBS controls (LCMV/PBS). (K) IFN‐α levels in serum of mice with or without HSV‐1 infection for 24 h (n = 3). (L) MFI of CCR2 and CCR5 on pDCs in the bone marrow of HSV‐1‐infected mice for 24 h (n = 3). (M) Percentage of splenic pDCs in mice during the HSV‐1 infection (n = 3). (N) Percentage of BM pDCs and mature pDCs (Sca‐1^+^ pDCs), and CD69 expression from the HSV‐1‐infected mouse model (5 × 10^6^ PFU/mouse) for 24 h (n = 3). (O) The relative expression of *Isg15* and *Mx1* in pDCs from bone marrow in the TMPD‐induced SLE mouse model for 2 weeks (500 µL TMPD/mouse, n = 3). (P) MFI of CCR2 and CCR5 on BM pDCs (n = 4). (Q) Percentage of splenic pDCs in the TMPD‐induced SLE mouse model (n = 4). (R) Percentage of BM pDCs and Sca‐1^+^ pDCs in the TMPD‐induced SLE mouse model (n = 4). Graphs represent the mean ± SEM. Symbols represent individual mice. Statistical significance was determined by one‐way ANOVA (A–D) or unpaired two‐tailed Student's *t*‐test (K–R) of n = 3 independent biological experiments. ^*^ *p* < 0.05, ^**^ *p* < 0.01, ^***^ *p* < 0.001, ^****^ *p* < 0.0001; ns, not significant.

Moreover, when we intraperitoneally infected mice with the LCMV‐ARM (Figure S15A), the proportion of splenic pDCs was significantly decreased in wild‐type mice (Figure S15B). Meanwhile, the proportion of pDCs in the BM slightly decreased, while the proportion of Sca‐1^+^ pDCs significantly increased (Figure S15B). Furthermore, CCR2 and CCR5 expression on BM pDCs was significantly reduced following the viral infection (Figure S15C,D), while the expression of *Slc44a2* remained unaffected (Figure S15D). In addition, elevated IFN‐α levels were detected in the BM extracellular fluid of infected mice (Figure S15E), but no detectable viral loads were observed in the BM, although the viral loads in the spleen were significantly increased (Figure S15F). To delineate the precise temporal window of feedback inhibition following LCMV infection, we performed a time‐course analysis of serum IFN‐α levels while concurrently monitoring the frequencies of BM and splenic pDCs, as well as the dynamic expression of CCR2, CCR5, and CD69 at multiple time points after infection (Figure 4F). We found that serum IFN‐α levels rose significantly and peaked at 24 h post‐infection (hpi), then returned to baseline by day 7 (Figure 4G). Correspondingly, we observed significant reductions at 24 hpi in: (1) CCR2 and CCR5 expression on BM pDCs (Figure 4H), (2) the proportion of splenic pDCs (Figure 4I), and (3) the frequency of BM pDC (Figure 4J). Conversely, the frequency of BM Sca‐1^+^ pDCs and CD69 expression on BM pDCs increased significantly from 24 hpi onward (Figure 4J). Notably, this inhibitory effect was transient; splenic pDC proportions, BM pDC frequencies, and CD69 expression returned to baseline by day 7, while CCR2/CCR5 expression and BM Sca‐1^+^ pDC frequencies recovered by day 12. Together, these results demonstrated that IFN‐I‐mediated feedback inhibition of BM pDC migration initiates at 24 hpi but progressively resolves from day 7, thereby balancing early antiviral immunity with the prevention of pathological IFN‐I overproduction.

Similarly, in mouse models of other viral infections (e.g., HSV‐1) and autoimmune diseases (e.g., SLE), pDC egress from the bone marrow was also suppressed (Figure 4L–N,P–R), accompanied by elevated systemic IFN‐I levels (Figure 4K,O and Figure S15G–I). Therefore, the enhanced IFN‐I production by viral infection or SLE suppressed *Ccr2* and *Ccr5* expression in BM pDCs.

### 2.5 Blocking IFN‐I Signaling Restores CCR2/CCR5 Expression, and pDC Egress in *Slc44a2*‐Deficient Mice

Based on the above results, we hypothesized that enhanced IFN‐I secretion by *Slc44a2‐*deficient pDCs restricted pDC migration from the BM to the periphery via suppressing the expression of CCR2 and CCR5. To further confirm this hypothesis in vivo, we administered an IFNAR1 antibody to cKO mice (Figure S16A), which efficiently blocked IFNAR1 (Figure S16B). Notably, CCR2 and CCR5 expression on *Slc44a2*‐deficient BM pDCs was significantly restored after IFNAR1 blockade (Figure S16C). Moreover, the proportion of splenic pDCs was also significantly increased, while no significant changes were observed in the inguinal lymph nodes and thymus (Figure S16D). Therefore, blocking IFNAR1 in cKO mice effectively restored the expression of CCR2 and CCR5 and the proportion of splenic pDCs. The less efficient recovery of pDCs in inguinal lymph nodes and thymus might be due to the relatively short duration of antibody treatment.

To further validate the impact of type I interferon signaling on the *Slc44a2*‐mediated pDC phenotype, we generated *Slc44a2* ^fl/fl^ *Ifnar1* ^fl/fl^ *Vav* ^iCre^ mice, which showed efficient deletion of IFNAR1 on BM pDCs (Figure 5A). Here, we observed a significant upregulation of CCR2 and CCR5 on BM pDCs from *Slc44a2*‐deficient mice upon *Ifnar1* deletion (Figure 5B). Additionally, the frequencies of pDCs in the spleen, inguinal lymph nodes, and thymus were significantly restored in *Slc44a2* ^fl/fl^ *Ifnar1* ^fl/fl^ *Vav* ^iCre^ mice, while the proportion of BM pDCs was markedly reduced (Figure 5C). Moreover, deletion of *Ifnar1* in *Slc44a2*‐deficient mice returned the hyper‐activated/matured BM pDCs to the baseline, represented by the down‐regulated expression of Sca‐1, CD69, ICAM‐1, KDR, CD40, CD80, CD86, and MHC‐II on BM pDCs (Figure 5D). Collectively, these results demonstrated that SLC44A2 modulates CCR2/CCR5 expression and BM pDC activation/maturation through an IFN‐I‐dependent signaling pathway.

**FIGURE 5:**
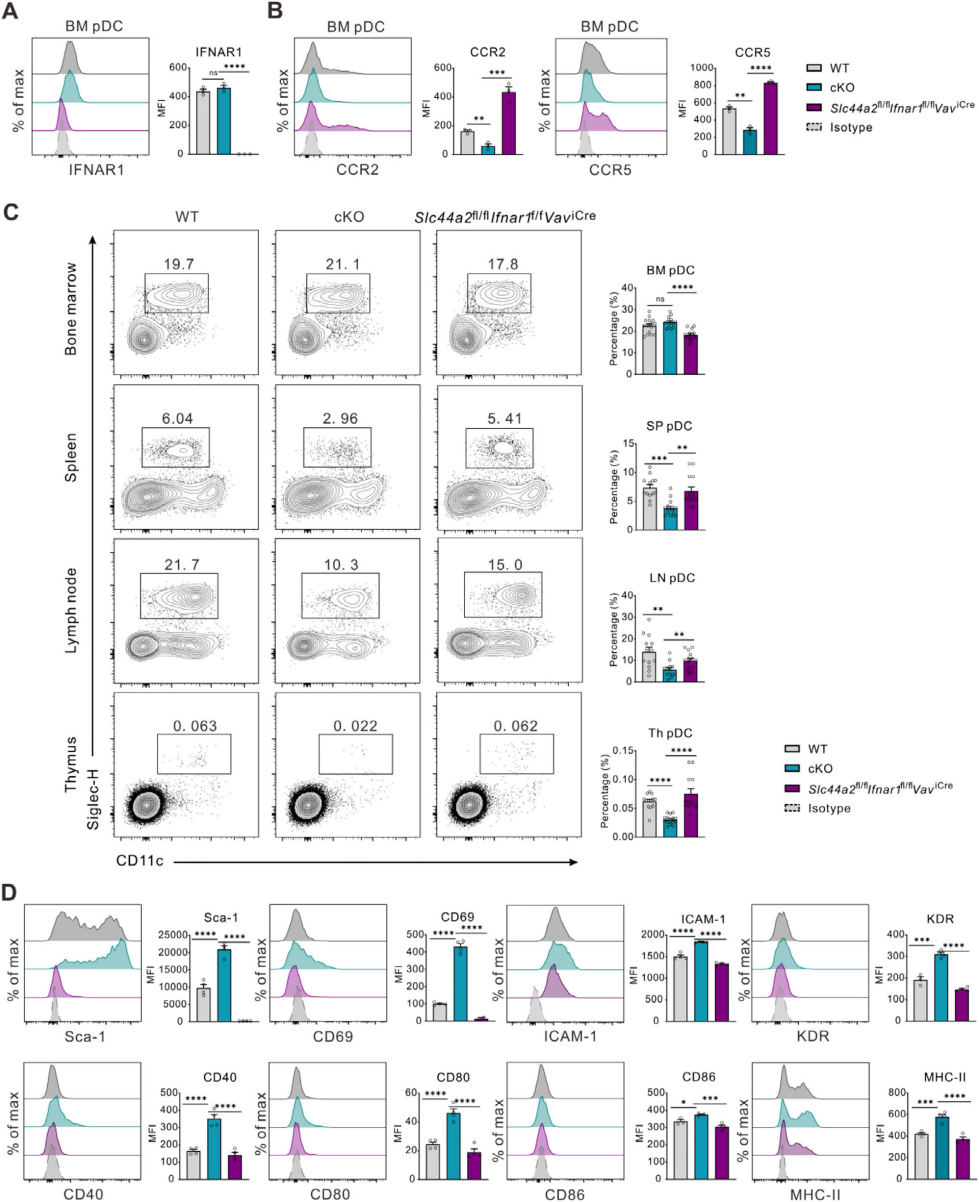
Blocking type I IFN signaling restores CCR2/CCR5 expression and pDC egress in *Slc44a2*‐deficient mice. (A,B) Flow‐cytometric analysis of IFNAR1, CCR2, and CCR5 expression on BM pDCs from WT, cKO, and *Slc44a2* ^fl/fl^ *Ifnar1* ^fl/fl^ *Vav* ^iCre^ mice (n = 3). (C) The percentage of pDCs in BM, SP, LN, and Th from WT, cKO, and *Slc44a2* ^fl/fl^ *Ifnar1* ^fl/fl^ *Vav* ^iCre^ mice (n = 12–15). pDCs were defined by live (7‐AAD^−^) CD45^+^ CD11b^−^CD3e^−^CD19^−^CD11c^int^ Siglec‐H^+^ cells. The percentage of pDCs in the BM, SP, LN, and Th was calculated as the proportion of CD11c^int^ Siglec‐H^+^ cells among live (7‐AAD^−^) CD45^+^ CD11b^−^ CD3e^−^ CD19^−^ cells. (D) The expression of Sca‐1, CD69, ICAM‐1, KDR, CD40, CD80, CD86, and MHC‐II in BM pDCs from WT, cKO, and *Slc44a2* ^fl/fl^ *Ifnar1* ^fl/fl^ *Vav* ^iCre^ mice was determined using flow cytometry (n = 4). Data were presented as mean ± SEM, with individual symbols representing individual mice. Statistical significance was determined using unpaired two‐tailed Student's *t*‐tests or one‐way ANOVA. ^*^ *p* < 0.05, ^**^ *p* < 0.01, ^***^ *p* < 0.001, ^****^ *p* < 0.0001; ns, not significant.

### 2.6 SLC44A2 Restricts IFN‐I Production by pDC via Regulating Intracellular Levels of Threonine, Asparagine, and Glutamine

To explore how SLC44A2 regulates IFN‐I production in pDCs, we first evaluated the role of choline and ethanolamine in this process, as both of them can be transported by SLC44A2 [17, 68]. In particular, blocking choline uptake significantly impacts ATP synthesis, mitochondrial membrane potential (MMP), and ROS production in mitochondria [27, 69]. This then activates the AMPK pathway [70]. Considering the essential role of AMPK‐regulated metabolic reprogramming in IFN‐α production by human pDCs [40], and the suppression of CCR2 expression by AMPK activation in RAW264.7 cells [71], it is likely that SLC44A2 regulates the function and migration of pDCs via the choline‐ATP‐AMPK pathway. However, in *Slc44a2‐*deficient BM pDCs, choline levels were significantly increased (Figure S17A), while ATP, MMP, and ROS generation showed no obvious change (Figure S17B–D). Accordingly, no significant impact on the AMPK pathway was detected (Figure S17E). Meanwhile, the expression of mitochondrial fusion proteins (MFN1 and MFN2) also showed no significant changes in *Slc44a2‐*deficient BM pDCs (Figure S17F). Lastly, administration of CPC in vivo, an AMPK inhibitor, did not change the phenotype of pDCs in cKO mice (Figure S17G), indicating relatively normal mitochondrial metabolism in pDCs of cKO mice. Consistently, the mTOR pathway remained largely intact in *Slc44a2*‐deficient pDCs, as evidenced by unchanged mTOR‐related transcriptional and proteomic profiles, together with comparable P‑mTOR (Ser2448) levels in BM pDCs (Figure S17H–J). These data suggest that the altered pDC function and migration in *Slc44a2*‐deficient mice may not be attributable to dysregulation of the choline‑AMPK axis or the canonical mTOR pathway. Likewise, various concentrations of choline and ethanolamine also showed no impact on the development and IFN‐α production by pDCs following CpG‐A stimulation (Figure S17K–M). Collectively, these data demonstrated that both choline and ethanolamine may not account for the disturbance of pDCs in *Slc44a2*‐deficient mice.

Therefore, SLC44A2 may regulate pDC homeostasis through additional, novel substrates. We next reanalyzed the metabolomic data of BM pDCs from WT and cKO mice. Notably, levels of several amino acids were significantly increased in cKO pDCs (Figure 6A), with highly enriched pathways such as amino acid transport and SLC transporter‐associated metabolic pathways (Figure 6B). Amino acid metabolism plays a crucial role in regulating cDC activation [32] and pDC function [72, 73]. Taurine can enhance IFN‐α secretion in pDCs by increasing IRF7 phosphorylation [72]. Additionally, amino acid transporters, such as SLC7A5, SLC3A2, and SLC7A11, are essential for human pDC activation [73]. To further investigate whether the increased amino acids in cKO pDCs were involved in IFN‐I production, we assessed changes in IFN‐α secretion following the depletion of the indicated amino acids. Notably, depletion of threonine (T), asparagine (N), or glutamine (Q) significantly reduced the ability of BM pDCs to secrete IFN‐α (Figure 6C). Loss of T, N, and Q in medium simultaneously led to a more profound reduction of IFN‐α production (Figure 6D). Conversely, increasing the concentrations of these amino acids significantly enhanced IFN‐α production (Figure 6E). Therefore, the intracellular concentrations of T, N and Q, which can be regulated by SLC44A2, determine the capacity for IFN‐α production in pDCs.

**FIGURE 6:**
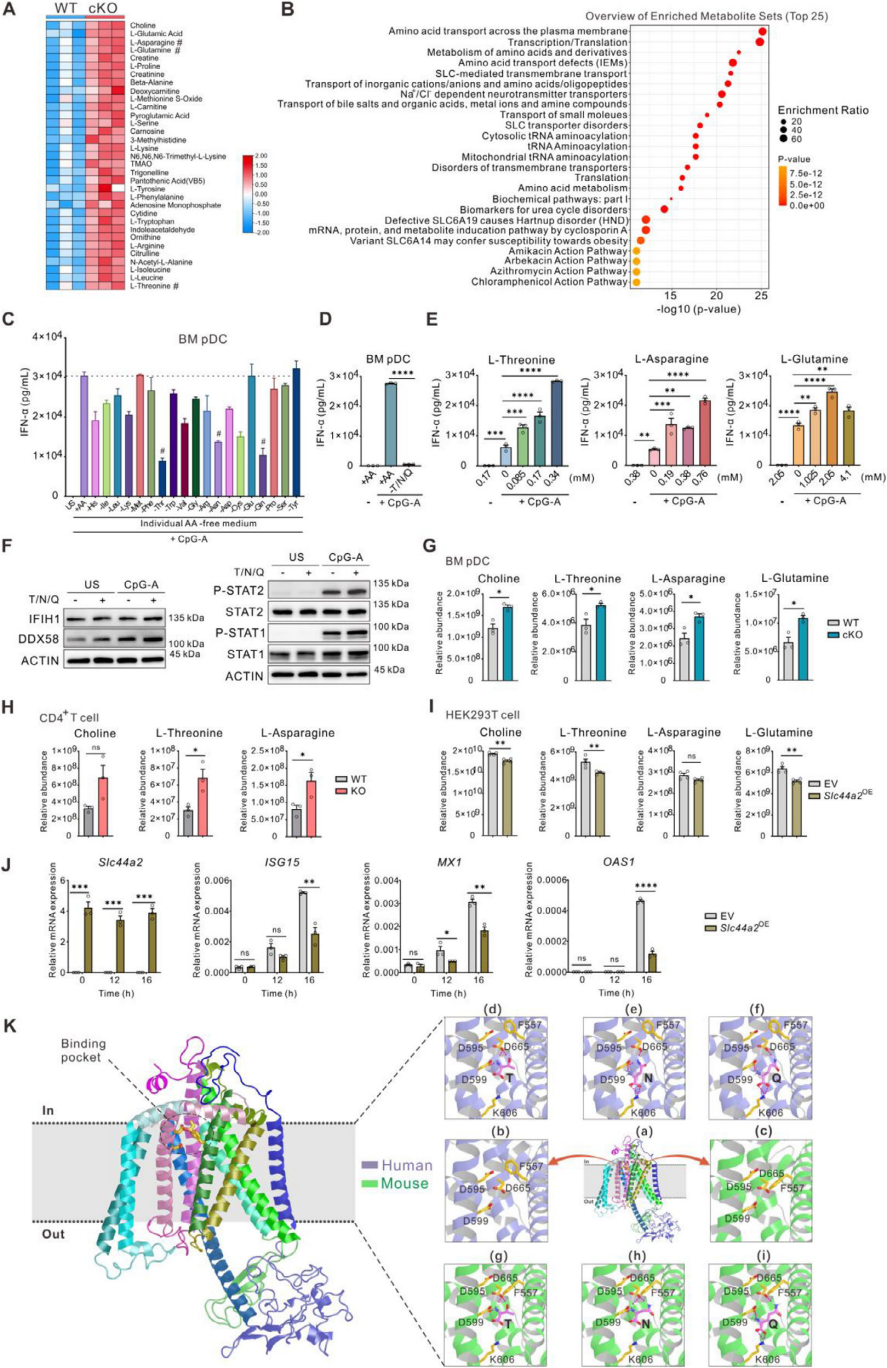
*Slc44a2* restricts IFN‐I production by pDCs via regulating intracellular levels of threonine (T), asparagine (N), and glutamine (Q). (A) Metabolic profiling of BM pDCs from WT and cKO mice. (B) Pathway enrichment analysis of significantly upregulated metabolites identified through metabolomics (n = 3). (C) Concentration of IFN‐α secreted by BM pDCs upon CpG‐A (1 µM) stimulation in vitro for 20 h in the absence of the indicated amino acid in the culture medium. US: unstimulated; AA: complete medium. (D) Concentration of IFN‐α secreted by BM pDCs upon CpG‐A (1 µM) stimulation in vitro for 20 h with or without threonine/asparagine/glutamine (abbreviated as T/N/Q) in the culture medium (n = 3). (E) Concentration of IFN‐α secreted by BM pDCs upon CpG‐A (1 µM) stimulation in vitro for 20 h with gradually increased concentrations of threonine (T), asparagine (N), or glutamine (Q) in the culture medium (n = 3). (F) Expression of IFIH1, DDX58, STAT1, P‐STAT1, STAT2, and P‐STAT2 in BM pDCs upon the stimulation of CpG‐A (1 µM) with or without three amino acids T/N/Q. US: unstimulated. (G) Quantification of choline, T, N, and Q in BM pDCs from WT and cKO mice (n = 3). (H) Quantification of choline, T, and N levels in CD4^+^ T cells from *Slc44a2* knockout mice (n = 3). (I) Quantification of choline, T, N, and Q in *Slc44a2*‐overexpressing HEK293T cells (n = 4). (J) HEK293T cells stably expressing empty vector (EV) or SLC44A2 (*Slc44a2* ^OE^) were stimulated with poly(I:C) (1 µg/mL) for the indicated times (0, 12, and 16 h). The mRNA levels of *Slc44a2*, *ISG15*, *OAS1*, and *MX1* were measured by qRT‐PCR and normalized to *β‐actin* (n = 3). (K) The potential binding pocket and residues in SLC44A2 with T, N, and Q in both human and mouse. (a) Overall structure of human and mouse SLC44A2, with membrane orientation predicted by the PPM 3.0 web server. The cytosolic side was indicated as “In,” and the extracellular or mitochondrial intermembrane space was labeled as “Out”. (b,c) Residues constituting the predicted largest‐volume binding pocket in human (light blue ribbons) and mouse (green ribbons) SLC44A2 were used to define the docking grid. (d–f) Close‐up view of human SLC44A2 bound to T, N, and Q, respectively. The polar interactions with the docked amino acids (magenta) and the binding site residues were shown as dashed violet lines. (h,i) Close‐up view of mouse SLC44A2 bound to T, N, and Q, respectively, with polar interactions similarly depicted. Data were presented as mean ± SEM, with individual data points indicated by symbols for each biological experiment. Statistical significance was determined by one‐way ANOVA (G–I) or unpaired two‐tailed Student's *t*‐test (D,E). ^*^ *p* < 0.05, ^**^ *p* < 0.01, ^***^ *p* < 0.001, ^****^ *p* < 0.0001; ns, not significant.

To further explore the mechanisms by which T/N/Q affect IFN‐I production in pDCs, we next assessed their effects on the classical amino acid‐sensing pathways mediated by mTOR and GCN2. Under unstimulated conditions, supplementation with T/N/Q enhanced phosphorylation of the mTOR pathway; however, no significant changes in mTOR signaling were observed upon CpG‐A stimulation (Figure S18A). This suggests that the marked reduction in IFN‐α production resulting from T/N/Q deficiency following CpG‐A stimulation is not mediated by the mTOR pathway. We also found that T/N/Q deficiency significantly increased phosphorylation of EIF2α, a downstream effector of GCN2, both under unstimulated conditions and after CpG‐A stimulation (Figure S18B). Nevertheless, treatment with an mTOR inhibitor or a GCN2 inhibitor did not restore the impaired IFN‐α secretion resulting from T/N/Q deficiency (Figure S18C). Taken together, these results suggested that the defect in IFN‐α production caused by T/N/Q deficiency is independent of both mTOR and GCN2 signaling pathways. Besides, we assessed the expression of IFIH1, DDX58, STAT1, P‐STAT1, STAT2, and P‐STAT2. The results revealed that depletion of T/N/Q significantly inhibited the expression of IFIH1, DDX58, P‐STAT1, and P‐STAT2 (Figure 6F), indicating that T/N/Q regulated IFN‐I production through the JAK‐STAT signaling pathway. However, the precise mechanism by which they potentiated STAT1/2 phosphorylation requires further investigation.

Correspondingly, *Slc44a2* knockout resulted in a significant increase in the intracellular levels of T, N, and Q in BM pDCs (Figure 6G). Similarly, T cell‐specific *Slc44a2* knockout led to a significant increase in the intracellular levels of both T and N (Figure 6H). Conversely, overexpression of *Slc44a2* in HEK293T cells resulted in a significant reduction in the levels of T and Q (Figure 6I). Moreover, poly(I:C)‐induced expression of the IFN‐stimulated genes, including *ISG15*, *OAS1*, and *MX1*, was markedly suppressed in HEK293T cells overexpressing *Slc44a2* at 16 h post‐stimulation (Figure 6J), indicating that SLC44A2 negatively regulates the type I interferon pathway. Thus, it is reasonable that SLC44A2 regulates the IFN‐α secretion of pDCs by modulating the intracellular concentrations of amino acids such as T, N, and Q.

Using the RosettaFold‐derived 3D‐structure models of human and mouse SLC44A2, we estimated the transmembrane (TM) segments and predicted the membrane orientation (Figure 6K) with the PPM 2.0 web server [74]. These analyses revealed that both proteins consist of 10 TM helices (Table S2). To identify the potential substrates for human and mouse SLC44A2 and explore the binding site interactions, we first performed a docking‐based virtual screening of the human metabolite library obtained from the Human Metabolome Database. Through screening, we identified 22 potential substrates related to mouse and human SLC44A2, including choline, glutamine, and aspartate, etc (Table S3 and S4). Notably, amino acids N, T, and Q, in their zwitterionic forms, bound to the predicted binding pocket in human and mouse SLC44A2 (Figure 6Ka–c). The positively charged amino group of these substrates formed significant hydrogen bond interactions with the negatively charged D599 and D665 within the predicted binding pocket (Figure 6Kd–f). Furthermore, the longer N and Q also formed additional interactions with the nearby K606 residue (Figure 6Ke,f). Human and mouse SLC44A2 exhibit high sequence identity (90.9%) and similarity (96.0%). Notably, the key residues F557, D595, D599, D665, and K606 in human SLC44A2 were also sequentially and structurally conserved in mouse SLC44A2 (Figure 6Kc,g–i), underscoring the functional conservation between the two orthologs. We observed similar interactions between these three amino acids and binding pocket residues. These results therefore suggested that SLC44A2 likely regulates the secretion of IFN‐I by pDCs via transporting T, N, and Q.

## 3 Discussion

In this study, we found that high expression of SLC44A2 in resting pDCs restricted IFN‐I production, possibly by exporting T, N, and Q, the amino acids essential for IFN‐I production. Meanwhile, enhanced IFN‐I production by pDCs due to *Slc44a2* deficiency restrained CCR2/CCR5‐mediated pDC egress. Accordingly, elevated IFN‐I levels during acute viral infection suppressed CCR2 and CCR5 expression and subsequent egress of BM pDCs. These findings reveal a critical role for SLC44A2 in regulating the function and egress of BM pDCs through the SLC44A2‐IFN‐I‐CCR2/CCR5 axis, which prevents uncontrolled systemic IFN‐I responses.

*Slc44a2*‐deficient mice exhibit a significant increase in splenic pDC apoptosis, whereas pDC apoptosis in lymph nodes and thymus remained unchanged (Figure S7B). Meanwhile, no differences in proliferation or apoptosis were observed in BM pDCs (Figure S7A,B). These findings suggest that the reduced peripheral pDC numbers in cKO mice are primarily attributable to impaired BM egress, with splenic apoptosis contributing to the local phenotype. Consistent with this, cKO mice exhibited significantly lower serum IFN‐α levels at early time points following CpG‐A stimulation, further supporting the notion that the reduced peripheral pDC pool limits systemic IFN‐I output under acute pDC‐centric challenge. To understand the molecular basis of the impaired egress, we examined the chemokine receptors and transcription factors known to regulate pDC migration. The egress of pDCs from the BM is a complex process involving various transcription factors and chemokine receptors, including RUNX2 and CXCR4 [6, 50, 51]. However, *Slc44a2*‐deficient pDCs showed no significant changes in these factors. Instead, our data suggest that decreased CCR2 and CCR5 expression accounted for the impaired pDC egress in *Slc44a2*‐deficient mice. Both receptors are crucial in mediating pDC migration [39, 42, 75], and CCR2 is well recognized for promoting the egress of multiple hematopoietic cell types from the bone marrow, including monocytes [76], and pre‐cDCs during viral infection [77], as well as DC migration and tissue localization in peripheral tissues [78]. Using *Ccr2* and *Ccr5* knockout mice, we found that CCR2 plays a more critical role than CCR5 in pDC egress, and that the two receptors function in a non‐redundant manner. Interestingly, the decreased *Ccr2* and *Ccr5* expression in *Slc44a2*‐deficient pDCs was driven by enhanced IFN‐I production. Similarly, elevated IFN‐I levels during viral infection suppressed CCR2 and CCR5 expression and restricted BM pDC migration. Consistent with these findings, human pDCs showed significantly decreased CCR2 and CCR5 expression following CpG‐A stimulation [79]. These results suggest that IFN‐I‐mediated suppression of *Ccr2* and *Ccr5* expression is a conserved mechanism restricting pDC egress from the BM.

Consistent with impaired pDC egress, we observed that mature Sca‐1^+^ pDCs accumulated in the BM of cKO mice. However, total BM pDC numbers did not increase substantially. This may reflect two counterbalancing effects of *Slc44a2* deficiency: the retention of mature pDCs due to CCR2/CCR5 downregulation and reduced de novo pDC production driven by elevated IFN‐I signaling, which is known to suppress pDC differentiation by downregulating the expression of E2‐2 [13]. Notably, neither *Ccr2*‐ nor *Ccr5*‐deficient mice exhibited significant accumulation of BM pDCs despite impaired egress (Figure 2I,J and Figure S9B,C), indicating that defective pDC egress does not invariably lead to proportional BM accumulation. Similarly, *Runx2*‐deficient and *DOCK2*‐deficient mice, both characterized by impaired pDC egress [6, 55], also show no significant increase in the percentages or numbers of total BM pDC despite peripheral reduction. Collectively, these observations suggest the existence of a homeostatic feedback mechanism whereby retained mature pDCs or their secreted factors may limit the differentiation of pDCs from progenitors such as CDPs [13], thereby constraining the overall BM pDC pool size.

Notably, Niederquell et al. demonstrated that Sca‐1^−^ pDCs produce more IFN‐α than Sca‐1^+^ pDCs upon endosomal TLR9 stimulation, and are thus considered the genuine natural IFN‐α‐producing cells in mice [41]. The enhanced IFN‐I‐producing capacity of Sca‐1^−^ pDCs has been linked to elevated expression of osteopontin (Opn) [41], a molecule essential for IFN‐α production [59, 60]. Interestingly, we observed that *Opn* expression was significantly upregulated in BM Sca‐1^+^ pDCs from *Slc44a2*‐deficient mice (Figure 3E), raising the possibility that loss of SLC44A2 promotes IFN‐α production at least partly by regulating *Opn* expression. Notably, among all type I and type III IFN genes examined, only *Ifna2* was significantly upregulated in cKO pDCs, suggesting a selective regulation of SLC44A2 on IFN‐I expression.

IFN‐I plays critical roles in immune responses. However, excessive IFN‐I production by over‐activated pDCs can lead to diseases such as infection‐associated pathology [11]. and autoimmune disorders (e.g., SLE) [8, 80]. Thus, IFN‐I production by pDCs must be tightly controlled to maintain immune homeostasis. Multiple sophisticated regulatory mechanisms governing this process have been identified, ranging from the regulation of IFN‐I expression to the control of pDC development, differentiation, and homeostasis. For instance, lactate dehydrogenase B (LDHB) acts as a positive regulator of IFN‐I production in pDCs; upon pDC activation, failure to downregulate LDHB expression leads to its sustained expression, which can trigger IFNAR‐dependent inflammatory diseases [11]. In this study, we confirmed that *Slc44a2* was highly expressed in resting pDCs but significantly decreased upon activation. Furthermore, *Slc44a2* deficiency enhanced IFN‐I production at steady state. Therefore, we propose SLC44A2 as a novel negative regulator of IFN‐I production in pDCs. Particularly, we found that SLC44A2 reduced the intracellular levels of three amino acids required for IFN‐I synthesis, T/N/Q, likely by exporting them. We further demonstrated that these three amino acids synergistically promote IFN‐I production by pDCs through the JAK‐STAT signaling pathway. Removal of these amino acids from pDC medium resulted in reduced IFN‐I production. Thus, SLC44A2 may function as a metabolic checkpoint that restricts spontaneous IFN‐I production by exporting T/N/Q from pDCs.

Several observations support an upstream role for T/N/Q in IFN‐I induction. Our data suggest that T/N/Q supplementation enhances IFN‐α production in the absence of exogenous IFN‐I (Figure 6E), and Asn (N) supplementation was recently shown to enhance the phosphorylation of TBK1, IRF3, and STAT1 in both HSV‐1‐ and VSV‐infected cells [81]. However, because IFN‐I signaling is intrinsically amplified through a positive‐feedback loop and *Ifih1*, *Ddx58*, and STAT1/2 phosphorylation are themselves IFN‐I‐inducible, their reduced expression in the absence of T/N/Q may be secondary to diminished IFN‐I production rather than direct upstream regulation. Thus, although autocrine IFN‐I amplification likely contributes to the observed effect, the direct molecular targets of T/N/Q remain unclear. Consistent with this, our data support an indirect mechanism whereby T/N/Q availability enhances IFN‐α production (Figure 6D), which subsequently suppresses *Ccr2* and *Ccr5* expression (Figure 4B,D).

Amino acid metabolism has been broadly involved in the regulation of interferon signaling. For example, tryptophan catabolism maintains the tolerogenic phenotype of conventional DCs via the IDO1‐kynurenine‐AhR axis and regulates immune tolerance primarily through type II interferon (IFN‐γ) [82]. Glutamine has been shown to modulate interferon responses in CD8^+^ T cells via the mTORC1 pathway [83]. Asparagine enhances IFN‐β production in macrophages and promotes STAT1 phosphorylation independently of the mTOR pathway [81, 84]. In this study, we identified T/N/Q as a distinct metabolic module that enhances type I interferon responses in pDCs, acting synergistically through the JAK‐STAT pathway in an mTOR‐independent manner. Thus, T/N/Q may function as a conserved metabolic module coupling nutrient availability to antiviral immunity across immune cell types.

Consistent with this, several lines of evidence support that SLC44A2 functions as a broadly conserved metabolic checkpoint calibrating IFN‐I responses across multiple immune cell types. First, phylogenetic analysis revealed that SLC44A2 orthologs are highly conserved across jawed vertebrates with functional type I interferon systems (Figure S2), suggesting an evolutionarily ancient role for this transporter in immune regulation. Second, in *Slc44a2*‐deficient pDCs, the intracellular levels of T, N, and Q were elevated, and this was accompanied by global upregulation of the type I interferon signaling pathway (Figure 3A–D). Third, we have also observed increased T/N/Q content in *Slc44a2*‐deficient T cells, and conversely, overexpression of SLC44A2 in HEK293T cells led to reduced intracellular T/N/Q levels and decreased ISG expression, demonstrating that SLC44A2‐mediated regulation of these amino acids and downstream IFN‐I responses is not restricted to pDCs. Fourth, ISGs were significantly upregulated in non‐pDC immune cells from the bone marrow and spleen of *Slc44a2*‐deficient mice at steady state (Figure S12A), indicating paracrine activation by pDC‐derived IFN‐I. Notably, this difference was no longer apparent following in vivo CpG‐A challenge (Figure S12B), suggesting that the ISG induction observed in non‐pDC cells at steady state is largely driven by chronically elevated IFN‐I from cKO pDCs. However, since *Vav‐iCre* also deletes *Slc44a2* in these non‐pDC cells, we cannot formally exclude a potential contribution from cell‐autonomous effects of *Slc44a2* deficiency in bystander cells. Regardless, these observations imply that IFN‐I can modulate the local hematopoietic niche through both autocrine and paracrine mechanisms, and further suggest that SLC44A2 may function as a broadly relevant metabolic checkpoint for IFN‐I responses across different immune cell types. Collectively, these findings support a model in which SLC44A2, by exporting T, N, and Q, acts as a metabolic gatekeeper that restricts excessive IFN‐I production not only in pDCs but potentially across multiple hematopoietic cell types.

The homeostasis of pDCs is maintained through multiple regulatory mechanisms operating at different levels. For instance, (1) IFN‐I signaling downregulates the expression of E2‐2, leading to impaired pDC development [13], and increased pDC apoptosis in the spleen [12], resulting in a markedly reduced number of pDCs in the periphery. (2) TNF‐α produced by activated pDCs suppresses TCF4 expression and promotes pDC reprogramming into cDC2, thereby preventing excessive production of IFN‐I [85]. (3) In this study, we demonstrated that IFN‐I restrained pDC egress from the BM by downregulating *Ccr2* and *Ccr5* expression. This feedback mechanism inhibited pDC egress under high IFN‐I conditions. Thus, IFN‐I, the principal cytokine product of pDCs, also serves as a feedback signal that triggers negative regulation at multiple stages of pDC biology, including development, migration, and survival. However, IFN‐I regulates immune cell function in a context‐dependent manner. IFN‐I upregulates CCR2 and downregulates CXCR4 on pre‐cDCs during viral infection, facilitating their egress from the BM [77]. The different regulation of pDCs and pre‐cDCs in the BM may stem from their distinct TLR profiles [86], suggesting that IFN‐I fine‐tunes the emigration of these subsets to maintain systemic immune homeostasis.

The findings presented here establish SLC44A2 as a key negative regulator of IFN‐I production in pDCs, acting likely by exporting T, N, and Q—a mechanism that may operate broadly across multiple immune cell types. The downregulation of *Slc44a2* upon pDC activation facilitates the metabolic demands of robust IFN‐I production. However, despite the enhanced per‐cell IFN‐I capacity observed in *Slc44a2*‐deficient pDCs, we did not detect overt disease exacerbation in models of viral infection or autoimmunity, suggesting that loss of *Slc44a2* alone is insufficient to drive systemic pDC overactivation and immunopathology. This likely reflects the redundancy of regulatory mechanisms that constrain pDC‐driven IFN‐I responses in vivo. Nevertheless, our observation that SLC44A2 overexpression suppresses ISG expression raises the possibility that enhancing the SLC44A2‐T/N/Q axis could represent a strategy to dampen aberrant IFN‐I responses therapeutically. In this regard, dietary supplementation with T/N/Q, or modulation of their availability through metabolic intervention, may offer a plausible approach to restrain excessive IFN‐I production in settings such as viral infection or SLE. Future studies are warranted to explore these possibilities.

## 4 Conclusion

In conclusion, our study reveals two interlinked mechanisms that fine‐tune pDC homeostasis and prevent aberrant immune activation (Figure S19). First, SLC44A2 functions as a negative regulator that restrains spontaneous IFN‐I production in pDCs. Second, an IFN‐I‐dependent feedback loop dynamically modulates pDC egress by regulating CCR2 and CCR5 expression. These two pathways likely act in concert to balance antiviral efficacy with immune restraint, ensuring the safety of pDC‐mediated immune responses.

## 5 Experimental Section

### 5.1 Mice

*Slc44a2* ^fl/fl^ mice were obtained from Cyagen Biosciences (Suzhou, China). The generation of these mice has been described previously [18]. *Vav‐iCre* mice were from Jackson lab (JAK stock: 008610) [87]. *Slc44a2* ^fl/fl^ *CD11c* ^Cre^ mice (C57BL/6J background) were generated by crossing *Slc44a2* ^fl/fl^ mice with *CD11c‐Cre* mice (JAK stock: 008068). Similarly, *Slc44a2* ^fl/fl^ mice were crossed with *Vav‐iCre* mice to produce *Slc44a2* ^fl/fl^ *Vav* ^iCre^ mice. *Ccr5* ^−/−^ (JAX stock: 005427) and *Ccr2* ^RFP/RFP^ (JAK stock: 017586) mice were kindly provided by Dr. Hai Qi and Dr. Xiaoyu Hu (Tsinghua University, China), respectively [88]. *Ccr5* ^−/−^ *Ccr2* ^RFP/RFP^ mice were generated by crossing *Ccr5* ^−/−^ mice with *Ccr2* ^RFP/RFP^ mice. *Slc44a2* ^fl/fl^ *Vav* ^iCre^ mice were further crossed with *Ifnar1* ^fl/fl^ mice to obtain *Slc44a2* ^fl/fl^ *Ifnar1* ^fl/fl^ *Vav* ^iCre^ mice. *Ifnar1* ^fl/fl^ mice were a gift from Dr. Yangxin Fu (Tsinghua University, China) [89]. For all experiments in this study, both age‐ and sex‐matched mice aged 6–12 weeks were used for experiments. All mice were bred and maintained in the specific pathogen‐free (SPF) grade animal facilities. The animal experiments were approved by the Institutional Animal Care and Use Committee of Tsinghua University.

### 5.2 Cell Line

HEK293T cells overexpressing *Slc44a2* were cultured in DMEM medium supplemented with 10% fetal bovine serum (FBS) and 1% penicillin‐streptomycin (Gibco) in a cell culture incubator (37°C, 5% CO_2_)_._ The cells were washed once with 2 mL of phosphate buffer saline (PBS, pH = 7.4), and then 1 mL of 0.05% trypsin‐EDTA was added for digestion at 37°C for 3 min. To neutralize the trypsin, 2 mL of DMEM containing FBS was added. After centrifugation at 1000 rpm for 3 min, the supernatant was removed, and the cells were collected for subsequent analysis.

### 5.3 Cell Suspension Preparation

Upon euthanasia of the mice, the femurs and tibias were harvested, and BM cells were flushed with PBS containing 3% FBS. The BM cells were exposed to red blood cell lysis buffer for 1 min, filtered through a 70 µm nylon cell strainer (Falcon), and centrifuged at 1750 rpm for 5 min at 4°C. After washing twice with PBS containing 3% FBS, BM cells were further enriched by negative selection using antibody‐containing cocktails (in‐house) and goat anti‐rat IgG beads (Bangs Laboratories) to remove CD3^+^, CD19^+^, CD11b^+^, Ly6G^+^, and TER‐119^+^ cells [90].

Spleen and inguinal lymph nodes were minced and digested with 1 mg/mL collagenase III (Worthington) and 0.1 mg/mL DNase I (Roche) in RPMI 1640 for 30 min at 37°C, followed by mechanical disruption through a 70 µm cell strainer. Red blood cells were lysed for 1 min using red blood cell lysis buffer for spleen samples. In parallel, thymic tissue was gently mashed through a 70 µm cell strainer in RPMI 1640. After washing and resuspension, 2 × 10^6^ cells from spleen, lymph nodes, and thymus were used for staining.

Peripheral blood was collected via retro‐orbital bleeding into EDTA‐coated tubes (BD Biosciences), with blood from three mice pooled as one biological replicate. One milliliter of blood was mixed with an equal volume of whole blood dilution buffer and carefully layered onto 3 mL of density gradient separation medium in a 15 mL tube. After centrifugation at 800 g for 25 min at room temperature, the mononuclear cell layer was collected and washed three times with PBS (250 g, 10 min, room temperature). The isolated cells were counted, and up to 2 × 10^6^ cells were used for staining.

Liver tissues were harvested after perfusion with PBS and minced. The tissue was homogenized in PBS containing 3% FBS using a tissue grinder, and the suspension was filtered through a 100 µm cell strainer. After centrifugation at 1750 rpm for 5 min at 25°C, the supernatant was discarded. The cell pellet containing immune cells was resuspended in 8 mL of 40% Percoll and centrifuged at 2500 rpm for 10 min at 25°C. The upper layer and interphase were carefully removed, and the cells were treated with red blood cell lysis buffer, washed twice with PBS (1750 rpm, 5 min, 4°C), and resuspended. Then, 2 × 10^6^ cells were used for staining.

### 5.4 Flow Cytometry Analysis

For surface staining, cells were pre‐incubated with anti‐mouse CD16/32 antibody (BioLegend) for 10 min at 4°C to block Fc receptors, then stained with appropriate antibody cocktails for 30 min on ice, protected from light. Antibodies against CD11c (N418), Siglec‐H (eBio440C), CD11b (M1/70), CD3e (145‐2C11), CD19 (6D5), CD24 (M1/69), CD45 (30‐F11), CD45.1 (A20), CD45.2 (104), CCR5 (HM‐CCR5), CD69 (H1.2F3), and Sca‐1 (D7) were purchased from eBioscience or BioLegend. CD172a (P84), CCR2 (475301), and P‐IRF7 (K47‐671) were purchased from BD Biosciences. Dead cells were excluded using 7‐AAD viability staining solution added 15 min before acquisition. For intracellular staining, BM pDCs were fixed and permeabilized using the Foxp3/transcription factor staining buffer set (eBioscience) after surface staining. The CD11c^int^ Siglec‐H^+^ pDC population was sorted using a FACSAria III system (BD Biosciences). Data were acquired on a flow cytometer and analyzed using FACSDiva software and FlowJo software (Tree Star).

### 5.5 Cell Culture and Stimulation

Sorted primary BM pDCs were incubated in complete medium: HEPES‐containing RPMI‐1640 basic medium (Gibco) supplemented with 20 ng/mL recombinant murine GM‐CSF (PeproTech), 20 ng/mL recombinant murine IL‐3 (PeproTech), 10% FBS, 1% 2‐mercaptoethanol (Gibco), and 1% penicillin‐streptomycin.

For FL‐DC induction, BM cells were cultured for 6–8 days in RPMI‐1640 medium supplemented with 10% FBS, 1% 2‐mercaptoethanol, 1% penicillin‐streptomycin, and 200 ng/mL Flt3L (PeproTech). For pDC stimulation, 2 × 10^5^ pDCs per well were seeded in 96‐well plates containing complete medium and stimulated with 1 µM CpG‐A (ODN2216, InvivoGen) for 24 h. The supernatant was then collected and stored at −20°C for subsequent ELISA assay. For IFN‐α treatment, BM‐derived pDCs were incubated with or without mouse IFN‐α (Miltenyi Biotec) for 24 h. To block IFN‐I signaling, *Invivo*MAb anti‐mouse IFNAR‐1 antibody (MAR1‐5A3, BioXCell) was used at the indicated time points.

For poly(I:C) stimulation, cells were seeded in 12‐well plates at 1 × 10^6^ cells per well in 1 mL of complete medium and cultured overnight to reach 60%–70% confluence. On the day of stimulation, cells from the 0 h time point were harvested directly in TRNzol. For the remaining wells, poly(I:C) (Sigma) was transfected using Hieff Trans LipoBooster 3000 (Yeasen) following the manufacturer's instructions and published protocol [91]. Briefly, a 1 µg/µL poly(I:C) working solution was prepared by diluting the 10 mg/mL stock in Opti‐MEM. Transfection complexes were formed by mixing LipoBooster 3000, poly(I:C), and Enhancer in Opti‐MEM according to the recommended ratio. After 15 min incubation, the culture medium was replaced with fresh complete medium, and the complexes were added dropwise to each well. Cells were harvested at 12 and 16 h post‐stimulation using TRNzol. All samples were stored at −80°C until RNA extraction.

### 5.6 ELISA

For IFN‐α detection in the supernatant, IFN‐α levels were measured using ELISA kits following the manufacturers’ instructions. Specifically, the VeriKine Mouse IFN‐α ELISA Kit (PBL Assay Science, Catalog #42120‐1) was used for experiments shown in Figure 3G. The LumiKine Xpress mIFN‐α 2.0 (InvivoGen, Catalog # luex‐mifnav2) was used for experiments shown in Figures 4G,K, 6C–E and Figures S11F,S13A,B,S14A–C,S15E,S17L,M, and S18C. To detect the IFN‐α in peripheral blood, 1 mL of blood from each mouse was collected and centrifuged at 3000 rpm for 15 min at 4°C to obtain serum, after allowing the blood to clot at room temperature for 1 h. The serum was then used for ELISA assay as described above. For IFN‐α measurement in the BM extracellular fluid [92], 500 µL of PBS was used to flush the femurs and tibias, and the supernatant was collected for further analysis after centrifugation at 1750 rpm for 5 min at 4°C.

### 5.7 RT‐qPCR

BM pDCs or tissues (liver and spleen) were lysed by TRNzol Universal (TIANGEN) to extract total RNA, which was then reverse transcribed into cDNA using the PrimeScript RT Reagent Kit with gDNA Eraser (Takara). RT‐qPCR was performed using Top Green qPCR SuperMix (TransStart) on the QuantStudio 7 Flex Real‐Time Fluorescence Quantitative PCR System (ABI). The threshold cycle (Ct) values of *β‐actin* were used to normalize the expression of target genes. The primers for LCMV gp in mouse were: CAT TCA CCT GGA CTT TGTCAG ACTC (Forward) and GCA ACT GCT GTG TTC CCG AAAC (Reverse) [34]. All primers used in this study are listed in Table S1.

### 5.8 RNA‐Seq Analysis

For RNA‐seq analysis, 2 × 10^6^ BM pDCs and BM Sca‐1^+^ pDCs were sorted from WT and cKO mice. pDCs were centrifuged at 1750 rpm for 5 min at 4°C to remove the supernatant. After resuspending in 1 mL of pre‐cooled PBS, the cells were centrifuged again, and the supernatant was discarded. To lyse the pDCs, 1 mL of TRNzol RNA extraction reagent (TIANGEN, DP424) was added, and the cells were thoroughly disrupted. Library construction and RNA sequencing were performed using the BGISEQ platform at the Beijing Genomics Institute (BGI, China). Differentially expressed genes were selected based on a *p‐*value < 0.05 and a |fold change| ≥ 1.5. The RNA‐seq data of BM pDC and BM Sca‐1^+^ pDCs from WT and cKO mice generated in this study have been deposited in the GEO database under accession numbers GSE326463 and GSE326459, respectively.

### 5.9 Proteomic Analysis

BM pDCs from WT and cKO mice were sorted by flow cytometry as described above. The sorted cells were then subjected to data‐independent acquisition (DIA)‐based proteomic analysis at the Proteomics Facility of Tsinghua University. Sample preparation and analysis were performed as previously described [93], with the following detailed parameters. Peptides were redissolved in solvent A (0.1% formic acid in water) and analyzed using an Orbitrap Exploris 480 mass spectrometer coupled to a Vanquish Neo UHPLC system (Thermo Fisher Scientific, MA, USA). Peptide samples were loaded onto a 15 cm analytical column (75 µm inner diameter, 1.9 µm resin) and separated with a 60‐min gradient. The gradient started at 4% buffer B (80% acetonitrile with 0.1% formic acid) for 5 min, followed by a stepwise increase to 35% over 41 min, then to 50% in 5 min, to 99% in 4 min, and held at 99% for 5 min. The flow rate was maintained at 300 nL/min with a column temperature of 55°C. The electrospray voltage was set to 2 kV.

For DIA experiments, full MS resolution was set to 120 000 at m/z 200, with an AGC target set to standard and an injection time in auto mode. The mass range was set to 400–1000 m/z. The AGC target for fragment spectra was set to 2500%. A total of 90 windows with a width of 6.7 Da were used. Fragment spectra resolution was set to 30 000 with an injection time of 22 ms. Normalized collision energy was set at 32%.

Raw DIA data were processed and analyzed using Spectronaut 16.0 (Biognosys AG, Switzerland) with default settings. Spectronaut was configured to search against the target database (version, entries), assuming trypsin as the digestion enzyme. Carbamidomethyl (C) was set as a fixed modification, and oxidation (M) as a variable modification. A Q‐value (FDR) cutoff of 1% was applied at both the precursor and protein levels. Normalization was performed using local normalization. The average of the top three filtered peptides that passed the 1% Q‐value cutoff was used to calculate protein abundance.

Differentially expressed proteins were identified using unpaired two‐tailed Student's *t*‐test, with selection criteria of *P* value < 0.05. The mass spectrometry proteomics data have been deposited to the ProteomeXchange Consortium (https://proteomecentral.proteomexchange.org) via the iProX partner repository [94]. with the dataset identifier PXD076360.

### 5.10 Western Blotting

The BM pDC extracts were prepared using the RIPA lysis buffer (Beyotime) containing a protease and phosphatase inhibitor cocktail (Roche). The protein concentration of the lysates was determined using the Pierce BCA Protein Assay Kit (Thermo Scientific) following centrifugation. The lysates were then denatured and subjected to SDS‐PAGE, followed by transfer onto PVDF membranes. Membranes were further blocked with skim milk (OXOID) and incubated with primary antibodies against target proteins. Primary antibodies against STAT1 (1:1000, 10144‐2‐AP) and β‐ACTIN (1:1000, 81115‐1‐RR) were purchased from Proteintech; STAT2 (1:1000, 72604S), P‐STAT1 (1:1000, 7649T), RIG‐1 (1:1000, 3743T), P‐AKT (Ser473) (1:1000, 4060T), AKT (pan) (1:1000, 4691T), P‐p70S6K (Thr389) (1:1000, 9234T), P‐p70S6K (Ser371) (1:1000, 9208T), p70S6K (1:1000, 34475T), P‐4E‐BP1 (Ser65) (1:1000, 9451T), P‐4E‐BP1 (Thr37/46) (1:1000, 2855T), 4E‐BP1 (1:1000, 9644T), P‐EIF2α (Ser51) (1:1000, 3398T), and EIF2α (D7D3) (1:1000, 5324T) were obtained from Cell Signaling Technology; P‐STAT2 (1:1000, 07–224) was from Merck Millipore. MDA5 (1:1000, A2419) was purchased from ABclonal. The HRP‐linked secondary antibodies, anti‐rabbit IgG (1:5000, 7074S) and anti‐mouse IgG (1:5000, 7076S), were obtained from Cell Signaling Technology.

### 5.11 Chemotaxis Assay

For in vitro migration assay, 2 × 10^5^ FACS‐purified BM pDCs in 100 µL basic medium (RPMI‐1640 + 1% FBS) were seeded in the upper well of a trans‐well chamber (Corning, 5 µm pore size), which had been pre‐coated overnight with 20 µg/mL collagen IV (Corning). Migration was assessed by allowing the pDCs to migrate toward the lower well, which contained 400 µL basic medium supplemented with chemokines RANTES (CCR5 ligand, 5 µg/mL) or MCP‐1 (CCR2 ligand, 5 µg/mL) (PeproTech). After 3 h of incubation at 37°C, pDCs that migrated to the lower well were harvested, and the cell number was determined by flow cytometry.

### 5.12 Mouse Treatment

For in vivo CpG‐A stimulation, WT and cKO mice were injected via the tail vein with 5 µg of CpG‐A (ODN2216, InvivoGen) complexed with 30 µL of DOTAP (MCE) as previously described [42, 95]. Serum IFN‐α levels were measured by ELISA kit at 6, 12, and 24 h post‐injection.

To block type I IFN signaling in vivo, cKO mice were injected intraperitoneally (i.p.) with 200 µg of IFNAR1‐neutralizing antibody (MAR1‐5A3, BioXCell) for five consecutive days, as described in previously established protocols [96]. Control mice received PBS treated in parallel. For in vivo IFN‐α treatment, mice were intravenously injected with 1.5 × 10^5^ U of mouse IFN‐α (Miltenyi Biotec) resuspended in 100 µL of sterile PBS or PBS alone, and were subsequently analyzed at 24 and 48 h [12].

For the TMPD (Pristane, MCE)‐induced IFN signature model, mice were i.p. injected with 500 µL TMPD for two weeks based on published protocols [97]. The size and weight of the spleen and kidney were analyzed. Besides, pDCs from bone marrow and spleen were collected for flow cytometric and RT‐qPCR analysis.

### 5.13 Virus Infection

For in vivo infection studies, mice were i.p. injected with 2 × 10^5^ plaque‐forming units (PFU) of LCMV‐ARM (a gift from Dr. Xuebin Liao) in a 200 µL volume, as per published methods [34]. The frequencies of pDCs in the BM and SP were assessed by FACS at indicated hours post‐infection. The liver and spleen were collected and stored at −80°C for subsequent RT‐qPCR analysis.

For the HSV‐1 infectious mouse model, mice were i.p. injected with 5 × 10^6^ PFU of HSV‐1 (a gift from Dr. Conggang Zhang) in a 200 µL sterile PBS according to the published methods [98]. Serum samples were collected for ELISA assay, and pDCs were isolated from the bone marrow and spleen for flow cytometric analysis after 24 h of infection.

### 5.14 Immunofluorescence Assay

The bones (femur and tibia), spleen, inguinal lymph nodes, and thymus tissues from WT and cKO mice were fixed overnight in 4% paraformaldehyde (LEAGENE). After fixation, the tissues were processed for embedding, sectioning, deparaffinization, antigen retrieval, and blocking. Sections of bone marrow, spleen, and inguinal lymph nodes were stained with antibodies specific for PDCA‐1 (Alexa Fluor 488, Green; Abcam) and B220 (eFluor 570, Red; Invitrogen) as previously described [51, 55]. Thymic sections were stained for PDCA‐1, where pDCs are known to localize in the thymic medulla [75]. The cortex (C) and medulla (M) were defined based on DAPI staining (Blue; Beyotime). Scale bars: 20 µm. Tissue sections were visualized using an Eclipse C1 confocal microscope (Nikon) and a Pannoramic MIDI slice scanner (3DHISTECH).

### 5.15 Bioinformatic Analysis

To identify SLC transporters involved in the specific metabolic regulation of pDCs, we preprocessed and performed quality control on RNA‐seq datasets of mouse (GSE109125) and human (GSE109348) immune cells during steady‐state conditions (non‐pathological or unstimulated) from the GEO database [99, 100]. This process involved filtering, normalization, and clustering. First, we performed a longitudinal screen to identify genes with expression levels exceeding the average expression of all genes in pDCs. Then, we conducted a cross‐sectional screen to identify differentially expressed genes with expression levels in pDCs ≥1.5 times those in other cell types. Subsequent Gene Ontology (GO) analysis further refined these genes to those involved in metabolic regulation. Next, we performed overlap analysis of the top 100 differentially expressed genes highly specific to pDCs in both mouse and human datasets, identifying two genes from the SLC family: *Slc44a2* and *Slc41a2*. We then revisited the differential gene lists for mouse and human pDCs, focusing specifically on all SLC family members. This analysis revealed five common SLC family genes shared by both species, with *Slc44a2* exhibiting the highest expression. Given that *Slc44a2* was predominantly recognized as a transporter involved in choline metabolism, we further examined the differential gene lists for genes related to choline metabolism in pDCs. Remarkably, *Slc44a2* remained the most highly expressed gene in both mouse and human pDCs.

### 5.16 Phylogenetic Analysis

SLC44A2 protein sequences for representative vertebrate species were retrieved from the NCBI Orthologs database (https://www.ncbi.nlm.nih.gov/gene/57153/ortholog/). Species included: *Homo sapiens* (human), *Mus musculus* (mouse), *Rattus norvegicus* (rat), *Myotis lucifugus* (bat), *Taeniopygia guttata* (zebra finch), *Anolis carolinensis* (green anole), *Thamnophis sirtalis* (garter snake), and *Danio rerio* (zebrafish). Multiple sequence alignment was performed using ClustalW as implemented in MEGA 12 (version 12.1.2) [101]. The phylogenetic tree was constructed using the neighbor‐joining method with the Jones‐Taylor‐Thornton (JTT) substitution model [102]. Evolutionary distances were computed in units of the number of amino acid substitutions per site, and the pairwise deletion option was applied to all ambiguous positions. Branch support was assessed by bootstrap analysis with 1000 replicates [103]. The tree was visualized and annotated using MEGA 12 and Adobe Illustrator. Species silhouettes were obtained from PhyloPic (https://www.phylopic.org) under the Creative Commons Attribution 3.0 Unported license and were colored according to taxonomic class for visual clarity.

### 5.17 Metabolic Analysis

BM pDCs were sorted from WT and cKO mice by flow cytometry as described above. For each sample, 2 × 10^6^ cells were collected, centrifuged to remove the supernatant, and resuspended in 1 mL of pre‐chilled 80% methanol (vol/vol) by vigorous pipetting. The samples were then stored at −80°C overnight to allow complete metabolite extraction. Samples were then centrifuged at 12 000 rpm for 10 min at 4°C. The supernatants were collected, dried under vacuum using a vacuum concentrator, and immediately sent to the Metabolomics and Lipidomics Center at Tsinghua University for LC‐MS/MS analysis.

Targeted metabolite screening was performed using a Synergi Hydro‐RP 100A column (Phenomenex, USA) with a column temperature of 35°C. The mobile phases consisted of A: water containing 2.376 mM tributylamine and 0.858 mM acetic acid, and B: methanol. The gradient elution was carried out at a flow rate of 250 µL/min as follows: 0–3.5 min, 1% B; 3.5–22 min, 1%–70% B; 22–23 min, 70%–90% B; 23–25 min, 90% B; 25–25.1 min, 90%–1% B; 25.1–30 min, 1% B. Data were processed using Tracefinder 3.2 (Thermo Fisher Scientific, USA). Metabolite identification was performed by matching MS/MS spectra against an in‐house library containing MS/MS spectra of over 1500 endogenous metabolites, with mass tolerances of 10 ppm for precursor ions and 15 ppm for fragment ions. Metabolites were assigned based on fragment matching, achieving two levels of identification: level 1 with MS/MS confirmation, and level 2 based on accurate precursor mass. Chromatographic peak areas were used for relative quantification, with a retention time shift of ± 0.25 min allowed for peak alignment.

To normalize metabolite abundance, the remaining cell pellets after methanol extraction were resuspended in 200 µL of 1 M KOH solution, vortexed thoroughly, and centrifuged at 12 000 rpm for 5 min at 4°C. The supernatants were collected, and protein concentration was determined using the BCA assay. Metabolite intensities were then normalized to protein concentration to account for differences in cell mass.

Differential metabolites between WT and cKO groups were identified using unpaired two‐tailed Student's *t*‐test with *p* < 0.05 as the cutoff criteria. The complete metabolomics dataset is provided in Table S5. Pathway enrichment analysis was performed using MetaboAnalyst 6.0 (https://www.metaboanalyst.ca/). Metabolites with *p* < 0.05 in the unpaired two‐tailed Student's *t*‐test were submitted to the Pathway Analysis module, with Mus musculus (KEGG) as the selected species. Enriched pathways were identified using the hypergeometric test for over‐representation analysis combined with relative‐betweenness centrality for topology analysis. Pathways with *p* < 0.05 were considered significantly enriched.

### 5.18 Modeling of Human and Mouse SLC44A2 Structures and Virtual Screening of Human Metabolites

The protein sequences for human and mouse SLC44A2 (UniProt IDs Q8IWA5 and Q8BY89, respectively) were retrieved from the UniProt database. These sequences served as inputs for structural modeling using RoseTTAFold [104] on the Robetta server [105]. Five 3D structure models were generated for each protein, and the top‐ranked model was selected based on its predicted local distance difference test (pLDDT) score. The selected protein structures were prepared using Protein Preparation Wizard [106]. In the Schrödinger software suite to ensure proper protonation states, bond orders, and geometry optimization, followed by energy minimization.

A comprehensive library consisting of human metabolites was downloaded from the Human Metabolome Database (HMDB). The metabolite structures were prepared using LigPrep, with the OPLS4 force field [107], and ionization states were generated at pH 7.4. The resulting metabolite library was subsequently docked against the energy‐minimized models of human and mouse SLC44A2 using Glide [108] in standard precision mode. Potential binding sites were first predicted using SiteMap [109], and docking was performed in the largest‐volume binding pocket with the default parameters. Top‐ranked metabolite candidates were determined by docking scores and subsequently filtered to retain only those with a molecular mass between 100 and 300 Da and a net charge ranging from −1 to +1. Additional inspection of docking poses was performed manually to identify substrate candidates, which were then experimentally validated.

### 5.19 Statistical Analysis

All data and graphs were analyzed using GraphPad Prism 8 (GraphPad Software) or Microsoft Excel. Results are presented as mean ± SEM, with individual data points representing biological replicates (individual mice). For comparisons between two groups, unpaired two‐tailed Student's *t*‐test was applied, while for comparisons involving more than two groups, one‐way ANOVA followed by Tukey's multiple‐comparisons test was used. Statistical significance was defined as ^*^ *p* < 0.05, ^**^ *p* < 0.01, ^***^ *p* < 0.001, ^****^ *p* < 0.0001; ns indicated not significant (*p* > 0.05).

## Author Contributions

Conceptualization: **Ligong Chen**, **Li Wu**, **Ruiqun Chen**, and **Tao Wu**. Methodology: Ruiqun Chen, Tao Wu, **Zhen Shi**, **Linlin Sheng**, **Lei Tao**, **Mingming Yang**, **Ravi Kumar Verma**, **Hao Fan**, **Conggang Zhang**, Ligong Chen, and Li Wu. Investigation: Ruiqun Chen, Tao Wu, Zhen Shi, Linlin Sheng, Mingming Yang, Ligong Chen, and Li Wu. Software: Ruiqun Chen, Tao Wu, Ravi Kumar Verma, Lei Tao, **Yuchen Wang**, and Hao Fan. Visualization: Ruiqun Chen, Tao Wu, Lei Tao, Ravi Kumar Verma, and Yuchen Wang. Funding acquisition: Ligong Chen, Li Wu, and Tao Wu. Supervision: Ligong Chen, Li Wu, Ruiqun Chen, and Tao Wu. Writing – review and editing: Ruiqun Chen, Tao Wu, Li Wu, and Ligong Chen.

## Conflicts of Interest

The authors declare no conflicts of interest.

## Supporting information




**Supporting File 1**: advs76325‐sup‐0001‐SuppMat.docx.

**Supporting File 2**: advs76325‐sup‐0002‐TableS1.xlsx.

**Supporting File 3**: advs76325‐sup‐0003‐TableS2.xlsx.

**Supporting File 4**: advs76325‐sup‐0004‐TableS3.xlsx.

**Supporting File 5**: advs76325‐sup‐0005‐TableS4.xlsx.

**Supporting File 6**: advs76325‐sup‐0006‐TableS5.xlsx.

**Supporting File 7**: advs76325‐sup‐0007‐Data.zip.

## Data Availability

The data that support the findings of this study are available from the corresponding author upon reasonable request.
