# Supplementary material for: Slc44a2 Deficiency Unveils an IFN‐I–Dependent Feedback Control of pDC Egress
Source: Adv Sci (Weinh). 2026 Jun 26;13(53):e76325. doi: 10.1002/advs.76325 (PMC13336407; doi:10.1002/advs.76325)
Supplement: Supplementary file 1 [file ADVS-13-e76325-s006.docx]

**Supplemental Figures**

**Figure S1.** *Slc44a2* is highly expressed in both human and mouse pDCs. A) Heatmap showing the genes that were highly expressed (fold change > 1.5, top 100) in both mouse and human pDCs, based on the published RNA-seq datasets. B) Heatmap showing the highly expressed genes belonging to the SLC family in mouse or human pDCs. C) SLC genes conservatively higher expressed between mouse and human pDCs. *Slc44a2* was highlighted with a red asterisk. D) Expression levels of *Slc44a2* among DC subsets from the spleen, data retrieved from the ImmGen database.

**Figure S2.** Evolutionary conservation of S*lc44a2* across species. A) Multiple sequence alignment of SLC44A2 protein sequences from representative vertebrate species (*Homo sapiens*, *Mus musculus*, *Rattus norvegicus*, *Myotis lucifugus*, *Taeniopygia guttata*, *Anolis carolinensis*, *Thamnophis sirtalis*, *Danio rerio*). B) Phylogenetic tree of SLC44A2 protein sequences from representative vertebrate species, constructed using the neighbor-joining method with the Jones-Taylor-Thornton (JTT) substitution model. Bootstrap values (1000 replicates) are indicated at the nodes; values represent the percentage of replicate trees in which the associated taxa clustered together, and are shown where support exceeds 65%. The tree is drawn to scale, with branch lengths measured in the number of amino acid substitutions per site. Species are color-coded by taxonomic class: mammals (purple), bird (orange), reptiles (yellow), and fish (blue). Silhouettes were obtained from PhyloPic (https://www.phylopic.org) under the Creative Commons Attribution 3.0 Unported (CC BY 3.0) license and were colored according to taxonomic class for visual clarity.

**Figure S3.** Deficiency of *Slc44a2* results in a significant reduction of pDCs in the peripheral tissues. A) The representative flow plots, proportions and cell numbers of pDCs in the bone marrow (BM), spleen (SP), inguinal lymph nodes (LN), and thymus (Th) of WT and cKO mice (n = 6-7). pDCs were defined by live (7-AAD^-^) CD45^+^ CD11b⁻CD3e⁻CD19⁻CD11c^int^ PDCA-1^+^ cells. B) Immunofluorescence analysis was conducted to visualize pDCs in the BM, SP, and LN of WT and cKO mice (n = 2). Sections of BM, SP, and LN, pDCs were stained with PDCA-1 (Alexa Fluor 488, Green) and B220 (eFluor 570, Red), while only PDCA-1 was used in the thymic sections. DAPI (Blue) was employed to distinguish the cortex (C) and medulla (M), with pDCs localized in the thymic medulla. Scale bar: 20 μm. Data were presented as mean ± SEM, with individual symbols representing individual mice. Statistical significance was determined using unpaired two-tailed Student’s t-tests. **p* < 0.05, ****p* < 0.001, *****p* < 0.0001; ns, not significant.

**Figure S4.** SLC44A2 is dispensable for the development of other major immune cells. A) Representative gating strategy of mouse spleen. Panel shows the gating strategy of CD11c^int^ Siglec-H^+^ pDCs, CD8α^+^ cDC1s, CD172a^+^ cDC2s, CD3e^-^ NK1.1^+^ NK cells, F4/80^+^ macrophage, CD19^+^ B cells, CD4^+^ T cells, and CD8^+^ T cells. B) Percentages of cDC1, cDC2, macrophages, NK cells, B cells, CD4^+^ T cells and CD8^+^ T cells in the BM, SP, and LN from WT and cKO mice (n = 6). Data were presented as mean ± SEM, with individual symbols representing individual mice. Statistical significance was determined using unpaired two-tailed t-tests of three independent biological experiments.. ***p* < 0.01; ns, not significant.

**Figure S5.** Peripheral pDCs are significantly reduced in *Slc44a2*^fl/fl^*CD11c*^Cre^ mice. A) Deletion efficiency of *Slc44a2* in FL-pDCs sorting from *Slc44a2*^fl/fl^*CD11c*^Cre^ mice (n = 4). B, C) Flow plots and percentages of pDCs in the BM, SP, LN and Th from *Slc44a2*^fl/fl^ and *Slc44a2*^fl/fl^*CD11c*^Cre^ mice, using the pDC-specific markers Siglec-H (B) and PDCA-1 (C) (n = 7-8). pDCs were defined by live (7-AAD^-^) CD45^+^ CD11b⁻CD3e⁻CD19⁻CD11c^int^ Siglec-H^+^ or PDCA-1^+^ cells. The percentage of pDCs in the BM, SP, LN, and Th was calculated as the proportion of CD11c^int^ Siglec-H^+^ cells among live (7-AAD^-^) CD45^+^ CD11b^-^ CD3e^-^ CD19^-^ cells. D) Percentages of cDC1, cDC2, macrophages, NK cells, B cells, CD4^+^ T cells and CD8^+^ T cells in the BM, SP, LN, and Th from *Slc44a2*^fl/fl^*CD11c*^Cre^ mice (BM, Th, n = 8; SP, LN, n = 16). Data were shown as mean ± SEM, with individual symbols representing individual mice. Statistical significance was determined using unpaired two-tailed Student’s t-tests of three independent biological experiments. **p* < 0.05, ***p* < 0.01, ****p* < 0.001; ns, not significant.

**Figure S6.** *Slc44a2* is dispensable for pDC development. A) Gating strategy for pDC, cDC1, and cDC2 derived from BM cells (FL-DC). B) Flow cytometry analysis of the impact of *Slc44a2* deletion (*Vav-iCre*) on the development of pDC, cDC1, and cDC2 (n = 7). The frequency of FL-pDCs was defined as the percentage of CD11c^int^ Siglec-H^+^ cells within the live (7-AAD^-^) cell population. The frequencies of FL-cDC1 and FL-cDC2 were defined as the percentages of CD24^+^ CD172a^-^ and CD172a^+^ CD24^-^ cells, respectively, within the live (7-AAD^-^) CD11c^+^ Siglec-H^-^ cell population. C) The representative flow plots, percentages and absolute numbers of FL-DC subsets derived from bone marrow cells of *Slc44a2*^fl/fl^ and *Slc44a2*^fl/fl^*CD11c*^Cre^ mice (n = 8). The frequency of FL-pDCs was defined as the percentage of CD11c^int^ Siglec-H^+^ cells within the live (7-AAD^-^) cell population. The frequencies of FL-cDC1 and FL-cDC2 were defined as the percentages of CD24^+^ CD172a^-^ and CD172a^+^ CD24^-^ cells, respectively, within the live (7-AAD^-^) CD11c^+^ Siglec-H^-^ cell population. Data were shown as mean ± SEM, with individual symbols representing individual mice. Statistical significance was assessed using unpaired two-tailed Student’s t-tests. ns, not significant.

**Figure S7.** Effect of *Slc44a2* knockout on proliferation and apoptosis of pDCs. A) Intracellular staining of Ki67 in BM pDCs from WT and cKO mice (n = 4). The percentages of Ki67^+^ pDCs was calculated as the proportion of Ki67^+^ pDCs within the pDC gate. B) Flow plots presenting the Annexin V and PI signals of pDCs in BM, SP, LN, and Th from WT and cKO mice (n = 5-6). Quadrant Q3 (Annexin V^+^/PI^-^) and Q2 (Annexin V^+^/PI^+^) represent early and late apoptotic pDCs, respectively. The percentages in quadrants Q3 (Annexin V^+^/PI^-^) and Q2 (Annexin V^+^/PI^+^) were calculated as the proportion of cells in each quadrant within the pDC gate. C) Apoptosis of FL-pDCs from WT and cKO mice (n = 11-12). The percentage of FL-pDCs was calculated as the proportion of CD11c^int^ Siglec-H^+^ cells among live (7-AAD^-^) cells. Data were shown as mean ± SEM, with individual symbols representing individual mice. Statistical significance was assessed using unpaired two-tailed Student’s t-tests based on n = 3 independent biological experiments. ns, not significant.

**Figure S8.** Enhanced features of maturation or activation of BM pDCs in *Slc44a2* deficient mice. A) Heatmap showing the selected differentially expressed genes related to pDC migration, maturation or activation in BM pDCs from WT and cKO mice (n = 3). B) Expression (FPKM values) of *Ly6a (Sca-1)*, *Cd69*, *Icam1*, *kdr*, *Cd40,* and *Cd86* in the RNA-seq data from BM pDCs of WT and cKO mice (n = 3). C) Flow cytometry analysis and MFI of Sca-1 on BM pDCs of WT and cKO mice (n = 6). D) Flow cytometry analysis of maturation- or activation-related molecules (ICAM-1, KDR, CCR9, CD40, CD80, CD86, and MHC-II) on BM pDCs from WT and cKO mice (n = 5-6). Data were shown as mean ± SEM, with individual symbols representing individual mice. Statistical significance was determined using unpaired two-tailed Student’s t-tests based on n = 3 independent biological experiments. ***p* < 0.01, ****p* < 0.001, *****p* < 0.0001; ns, not significant.

**Figure S9.** Inhibition or knockout of *Ccr5* and *Ccr2* results in significant reduction of pDCs in peripheral tissues. A) Schematic diagram of CCR5 and CCR2 inhibitor treatment. WT mice were gavaged with either solvent (vehicle) or a dual inhibitor of CCR5 and CCR2 (CVC). Mice were dosed at 50 mg/kg/day for 3 consecutive days. Representative flow plots and percentages of pDCs in the BM, SP, LN, and Th were shown (n = 4). pDCs were defined by live (7-AAD^-^) CD45^+^ CD11b⁻CD3e⁻CD19⁻CD11c^int^ Siglec-H^+^ cells. B) The cell ratio and numbers of pDCs in the BM, SP, LN, and Th of *Ccr5*^-/-^ knockout mice (n = 5). pDCs were defined by live (7-AAD^-^) CD45^+^ CD11b⁻CD3e⁻CD19⁻CD11c^int^ Siglec-H^+^ cells. C) The percentages and quantities of pDCs in the BM, SP, LN, and Th of *Ccr2* knockout mice (*Ccr2^RFP/RFP^*) (n = 5). pDCs were defined by live (7-AAD^-^) CD45^+^ CD11b⁻CD3e⁻CD19⁻CD11c^int^ Siglec-H^+^ cells. D) Flow cytometry analysis of the deletion efficiency of *Ccr5* in *Ccr5*^-/-^ knockout mice (n = 5). E) Representative genotyping plots for *Ccr2*^RFP/RFP^ knockout mice. The WT allele was 494 bp, and the RFP allele was 320 bp. F) MFI of CCR2 expression on BM pDCs from *Ccr5*^-/-^ knockout mice (n = 5). G) Flow plots, proportions and absolute numbers of pDCs in the BM, SP, LN, and Th of *Ccr5*^+/-^ *Ccr2*^RFP/+^ mice (n = 6). pDCs were defined by live (7-AAD^-^) CD45^+^ CD11b⁻CD3e⁻CD19⁻CD11c^int^ Siglec-H^+^ cells. Data were presented as mean ± SEM, with individual symbols representing individual mice. Statistical significance was determined using unpaired two-tailed Student’s t-tests based on n = 3 independent biological experiments. **p* < 0.05, ***p* < 0.01, ****p* < 0.001, *****p* < 0.0001; ns, not significant.

**Figure S10.** *Slc44a2* deletion shows no obvious effect on CXCR4, CXCR3, or CCR7-mediated migration, cytoskeletal dynamics, cell motility, or adhesion molecules expression in BM pDCs. A, B) The mRNA (A) and protein levels (B) of RUNX2 in BM pDCs from WT and cKO mice (n = 3). C, D) Flow cytometry analysis of CXCR4 expression (C, n = 6) on BM pDCs derived from WT and cKO mice, and their response to CXCL12 (100 ng/mL) during the transwell migration assay (D, n = 3-6). E, F) MFI of CXCR3 expression (E, n = 5) on BM pDCs from WT and cKO mice was analyzed by flow cytometry, and the transwell assay (F, n = 3-5) in response to CXCL9 (1 μg/mL). G) RNA-seq analysis of *Ccr7* expression in BM pDCs from WT and cKO mice (n = 3). H) Transwell analysis of BM pDCs from WT and cKO mice in response to CCL21 (30 nM, n = 1-4). I) Immunofluorescence analysis of F-actin polymerization in sorted BM pDCs from WT and cKO mice after stimulation with CCL21 (100 ng/mL) at various time points. F-actin was stained with Phalloidin-FITC, and nuclei were stained with DAPI. Images were captured using a Leica SP8 STED super-resolution confocal microscope (100× oil immersion objective). J) Chemotaxis assay of BM pDCs from WT and cKO mice using the EZ-TaxiScan system. CCL21 (250 μg/mL) was added to the upper wells of the chip, and BM pDCs (2×10^5^ cells/well) were placed in the lower wells. Chemotactic movement was recorded in real-time over 3 hours, with images taken every 30 seconds. K) MFI of ICAM-1 on pDCs in the BM, SP, LN, and Th from WT and cKO mice (n = 5). L) Expression of adhesion molecules CD11c, CD11a, CD18, and CD49d in sorted BM pDCs from WT and cKO mice, as measured by flow cytometry (n = 5). Data were presented as mean ± SEM, with individual symbols representing individual mice. Statistical significance was determined using unpaired two-tailed Student’s t-tests based on n = 3 independent biological experiments. **p* < 0.05, ***p* < 0.01, ****p* < 0.001, *****p* < 0.0001; ns, not significant.

**Figure S11.** *Slc44a2*-mediated pDC activation under steady-state condition is MyD88 independently. A) Schematic diagram of RNA-seq analysis of BM pDCs. B) Expression (FPKM) of *Irf7* in BM pDCs from WT and cKO (n= 3). C) Flow cytometry analysis of IRF7 expression in BM pDCs from WT and cKO mice (n = 3-4). D) Heatmap showing the increased expression of genes associated with the IFN-I signaling pathway (n = 3). E) The expression (FPKM) of type I and type III interferon genes in BM Sca-1^+^ pDCs from WT and cKO mice (n = 3). F) Expression (FPKM value) of *Opn* in BM Sca-1^+^ pDCs of WT and cKO mice (n = 4). G) The mRNA expression (FPKM) of *Tlr7*, *Tlr9*, *Myd88*, *Irak1*, *Irak4*, and *Traf6* from RNA-seq data in BM pDCs from WT and cKO mice (n = 3). H) The expression levels of TLR7, TLR9, MyD88, IRAK1, IRAK4, and TRAF6 in the DIA-based proteomics of BM pDCs from WT and cKO mice (n = 3). I) Flow cytometric analysis of Sca-1, KDR, ICAM-1, MHC-II and CD40 on WT and cKO BM pDCs treated with ST2825 (20 μM) or DMSO for 24 hours (n =3). J) Expression (FPKM) of *Ifnar1, Ifnar2, Jak1, Jak2, and Jak3* in RNA-seq dataset of BM pDCs from WT and cKO mice (n = 3). Data were presented as mean ± SEM, with individual symbols representing an biological sample. Statistical significance was determined using unpaired two-tailed Student’s t-tests. **p* < 0.05, ***p* < 0.01, ****p* < 0.001, *****p* < 0.0001; ns, not significant.

**Figure S12.** ISG expression in non-pDC immune cells from WT and cKO mice at steady state and after CpG-A challenge. A) RT-qPCR analysis of *Slc44a2* and the indicated ISGs (*Isg15, Mx1, Cxcl10, Oas1a, Ifit1*) in FACS-sorted CD45^+^ CD11c^-^ Siglec-H^-^ cells from the bone marrow and spleen of WT and cKO mice at steady state (n = 3). B) RT-qPCR analysis of the same genes in non-pDC cells 6 hours after intravenous injection of CpG-A (5 μg CpG-A + 30 μg DOTAP) *in vivo* (n = 4). Data were presented as mean ± SEM, with individual symbols representing individual biological replicates. Statistical significance was determined using unpaired two-tailed Student’s t-tests. **p* < 0.05, ***p* < 0.01, ****p* < 0.001, *****p* < 0.0001; ns, not significant.

**Figure S13.** *Slc44a2* knockout shows no significant influence on the systemic IFN-I levels or antiviral responses. A) Serum IFN-α levels in WT and cKO mice under homeostasis and CpG-A stimulation (5 μg/mouse) at 6, 12, and 24 hours (n = 3-4). B, C) WT and cKO mice were intraperitoneally injected with LCMV-ARM (2×10^5^ PFU/mouse). Blood, liver, and spleen were collected at different time points. IFN-α levels (B) and viral loads (C) were measured by ELISA and RT-qPCR, respectively (n = 3-4). D) The size and weight of spleen and kidney after 2 weeks of TMPD or mineral oil treatment (n = 2-3). Data were presented as mean ± SEM, with individual symbols representing individual mice. Statistical significance was assessed using unpaired two-tailed Student’s t-tests. ns, not significan.

**Figure S14.** Type I IFN signaling negatively regulates CCR2 and CCR5 expression in BM pDCs. A) IFN-α levels in BM pDCs stimulated with CpG-A (1 μM) *in vitro* for 0, 3, 6, 12, and 20 hours, measured by RT-qPCR. B) Expression of *Ccr2*, *Ccr5*, and *Slc44a2* in BM pDCs at different time points following CpG-A (1 μM) stimulation *in vitro*. C) *Ifna* mRNA levels in BM pDCs and IFN-α production by BM pDCs in the culture supernatants after treatment with CpG-A (1 μM) accompanied by different concentrations (0, 2, 25, 50 μg/mL) of IFNAR1 neutralizing antibody (anti-IFNAR1) for 8 hours (n = 3). D) Expression of *Ccr5*, *Ccr2*, and *Slc44a2* in BM pDCs treated with CpG-A (1 μM) accompanied with different concentrations of anti-IFNAR1 antibody (n = 3). Data were expressed as mean ± SEM, with individual data points denoted by symbols representing biological repeats. Statistical significance was determined by one-way ANOVA. **p* < 0.05, ***p* < 0.01, *****p* < 0.0001; ns, not significant.

**Figure S15.** Inhibition of pDC egress from BM during LCMV infected and SLE mouse model. A) Experimental schematic diagram of LCMV infection: mice were intraperitoneally injected (i.p.) with LCMV virus (2×10^5^ PFU/mouse) for 72 hours. Bone and spleen were isolated for the following analysis. B) Flow cytometric analysis of pDCs, Sca-1^+^ pDCs and SP pDCs in WT mice treated with PBS or LCMV for 72 hours (n = 5). The percentage of pDCs in BM and SP was calculated as the proportion of CD11c^int^ Siglec-H^+^ cells among live (7-AAD^-^) CD45^+^ CD11b⁻CD3e⁻CD19^-^ cells. The percentage of BM Sca-1^+^ pDCs was calculated as the proportion of Sca-1^+^ cells within the CD11c^int^ Siglec-H^+^ cells. C) Relative MFI of CCR2 and CCR5 on BM pDCs of WT mice treated with PBS or LCMV (n = 5). D) mRNA levels of *Ccr5*, *Ccr2*, and *Slc44a2* in isolated BM pDCs from WT mice treated with PBS or LCMV, measured by RT-qPCR (n = 3). E) IFN-α production in the BM extracellular fluid of WT mice treated with PBS or LCMV, quantified by ELISA (n = 13). F) RT-qPCR analysis of viral loads (LCMV gp) in BM and SP cells from LCMV-infected WT mice (n = 5). G) The size of spleen in the TMPD-induced SLE mouse model for 2 weeks (500 μL TMPD/mouse, n = 3). H) The relative expression of *Isg15*, *Mx1*, and *Ifit1* in splenic pDCs (n = 3). I) MFI of CCR2 and CCR5 on splenic pDCs in the TMPD-induced SLE mouse model (n = 4). Data were expressed as mean ± SEM, with individual data points denoted by symbols representing individual mice or biological repeats. Statistical significance was determined by unpaired, two-tailed Student’s t-test. **p* < 0.05, ***p* < 0.01, ****p* < 0.001, *****p* < 0.0001; ns, not significant.

**Figure S16.** Blocking IFN-I signaling restored CCR2 and CCR5 expression, partially rescuing pDC defects in *Slc44a2*-deficient mice. A) Experimental schematic diagram of anti-IFNAR1 antibody treatment *in vivo*. Mice were administered an intraperitoneal injection of either anti-IFNAR1 (200 μg/mouse) or PBS daily for five days, and pDCs from BM, SP, LN, and Th were analyzed. B) MFI of IFNAR1 on WT BM pDCs when treated with either PBS or anti-IFNAR1 (n = 3). C) Flow cytometry analysis and MFI of CCR2 and CCR5 on BM pDCs from WT and cKO mice treated with either PBS or anti-IFNAR1 antibody (n = 5-6). D) The flow plots, percentages and absolute numbers of pDCs in the BM, SP, LN, and Th from WT and cKO mice treated with PBS or anti-IFNAR1 antibody (n = 4-6). pDCs were defined by live (7-AAD^-^) CD45^+^ CD11b⁻CD3e⁻CD19⁻CD11c^int^ Siglec-H^+^ cells. Data were expressed as mean ± SEM, with individual data points denoted by symbols representing individual mice. Statistical significance was determined by one-way ANOVA. **p* < 0.05, ***p* < 0.01, ****p* < 0.001, *****p* < 0.0001; ns, not significant.

**Figure S17.** *Slc44a2* deficiency does not affect mitochondrial metabolism and mTORC1 signaling in BM pDCs. A) The relative abundance of choline in the BM pDCs from WT and cKO mice was determined by metabolomics analysis (n = 3). B) The ATP generation in the BM pDCs of WT and cKO mice (n = 5). C) The mitochondrial membrane potential (MMP) in BM pDCs isolated from WT and cKO mice (n = 5-6). D) Reactive oxygen species (ROS) assay in BM pDCs from WT and cKO mice (n = 5). E) Western blot analysis of AMPK and phosphorylated AMPK (P-AMPK) expression in BM pDCs from WT and cKO mice. F) The expression of mitochondrial fusion proteins MFN1 and MFN2 in BM pDCs from WT and cKO mice was detected using western blot. G) The CCR2 and CCR5 expression, and proportion of pDCs in the BM, SP, LN, and Th were analyzed by flow cytometry after WT and cKO mice were treated with AMPK inhibitor CPC or control solvent (n = 5). pDCs were defined by live (7-AAD^-^) CD45^+^ CD11b⁻CD3e⁻CD19⁻CD11c^int^ Siglec-H^+^ cells. H) The expression levels (FPKM) of mTOR pathway-related genes in RNA-seq analysis from WT and cKO mice (n = 3). I) The expression levels (relative abundance) of mTOR pathway-related proteins in the DIA-based proteomics of BM pDCs from WT and cKO mice (n = 3). J) The P-mTOR (Ser2448) expression in BM pDCs from WT and cKO mice was analyzed by flow cytometry (n = 5). K) The percentage of FL-pDCs with the treatment of varying choline concentrations during the Flt3L-mediated DC generation *in vitro* (n = 3). The percentage of FL-pDCs was calculated as the proportion of CD11c^int^ Siglec-H^+^ cells among live (7-AAD^-^) cells. L) Concentration of IFN-α secreted by BM pDCs treated with different doses of choline upon CpG-A stimulation were measured by ELISA (2 × 10^5^ pDCs/well, CpG-A: 1 μM, stimulated for 24 hours, n = 3). M) The effects of various concentrations of ethanolamine on IFN-α secretion by BM pDCs (2 × 10^5^ pDCs/well, CpG-A: 1 μM, stimulated for 24 hours, n = 3). Data were shown as mean ± SEM, with individual symbols representing individual mice or biological repeats. Statistical significance was determined using unpaired two-tailed Student’s t-tests. **p* < 0.05, ***p* < 0.01, ****p* < 0.001, *****p* < 0.0001; ns, not significant.

**Figure S18.** mTOR and GCN2 signaling are dispensable for the reduction of IFN-α production caused by the lack of T/N/Q. A) Western blot analysis of the expression of P-AKT (S473), AKT, P-p70S6K (T389), P-p70S6K (T371), P70S6K, P-4E-BP1 (Ser65), P-4E-BP1 (Thr37/46), and 4E-BP1 in BM pDCs cultured in complete medium versus T/N/Q-deficient medium following 2.5 hours CpG-A (1 μM) stimulation. US: unstimulated. B) Western blot analysis of the expression of EIF2α and phospho-EIF2α (P-EIF2α) in BM pDCs cultured in complete medium versus T/N/Q-deficient medium following 2.5 hours CpG-A (1 μM) stimulation. US: unstimulated. C) IFN-α concentrations in the supernatants of BM pDCs cultured under the indicated medium conditions (w/o T/N/Q) and pretreated with the rapamycin (0.1 μM) and GCN2 inhibitor GCN2iB (5 μM) for 3 hours, and then stimulated with CpG-A (1 μM) for 20 hours (n = 3). US: unstimulated. Data were presented as mean ± SEM, with individual symbols representing individual biological repeats. Statistical significance was determined using unpaired two-tailed Student’s t-tests. ***p* < 0.01, ****p* < 0.001.

**Figure S19.** Working model of SLC44A2-mediated maintenance of pDC homeostasis. This model illustrates two central mechanisms by which SLC44A2 regulates pDC homeostasis: (1) SLC44A2 limits IFN-I production by exporting amino acids (T, N, Q), thereby preventing spontaneous pDC activation. (2) Environmental IFN-I sensing controls pDC migration: Elevated IFN-I levels downregulate CCR2 and CCR5 expression, impairing pDC egress from the bone marrow to peripheral tissues. Together, these pathways prevent systemic IFN-I hyperresponse and maintain pDC functional balance.
