# Supplementary material for: Slc44a2 Deficiency Unveils an IFN‐I–Dependent Feedback Control of pDC Egress
Source: Adv Sci (Weinh). 2026 Jun 26:e76325. Online ahead of print. doi: 10.1002/advs.76325 (PMC13336407; doi:10.1002/advs.76325)
Supplement: Supplementary file 7 — Supporting File 7: advs76325‐sup‐0007‐Data.zip. [file ADVS-9999-e76325-s004.zip › 20260612_The raw, unedited immunoblots presented in the figures (9979792).pdf]

The raw, unedited immunoblots presented in the figures

Source of Figure. 3H

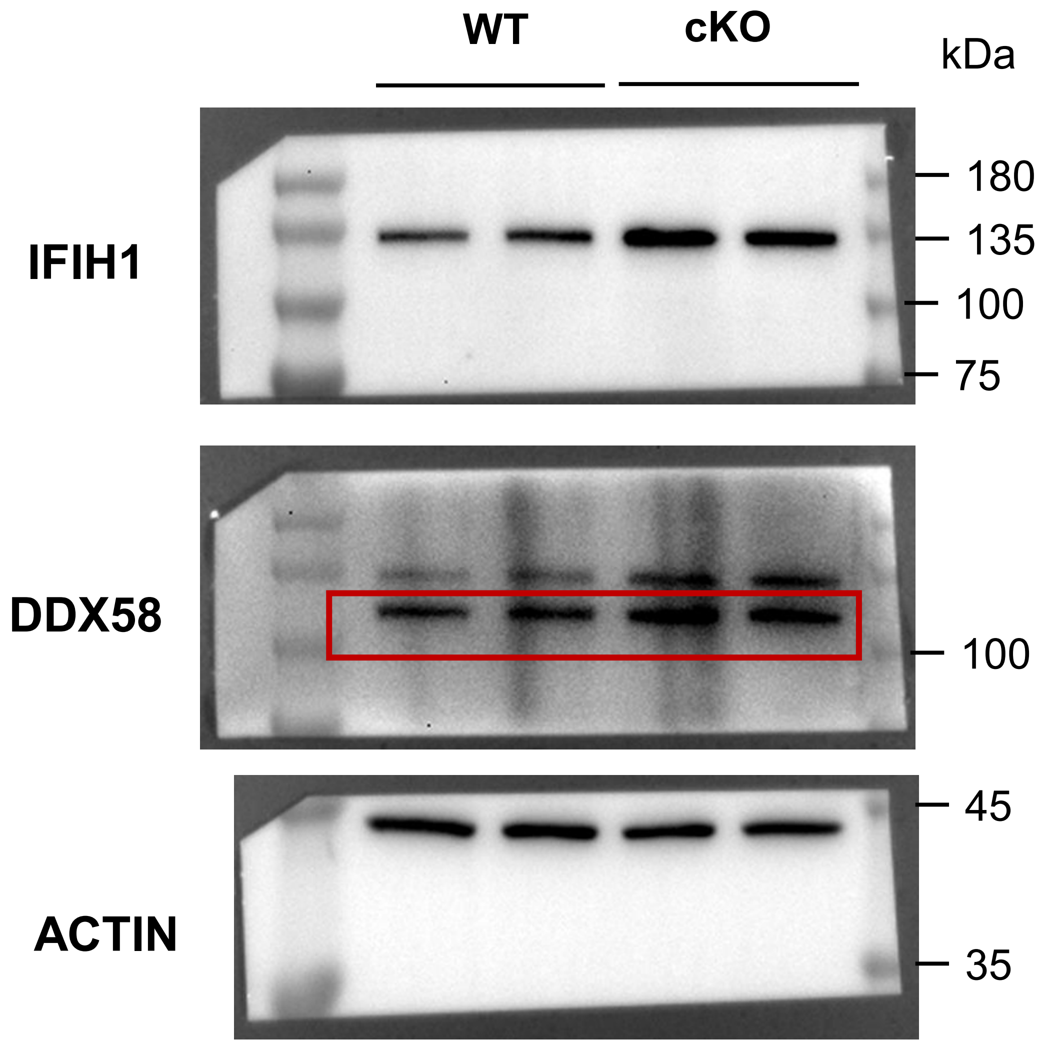

Source of Figure. 3I

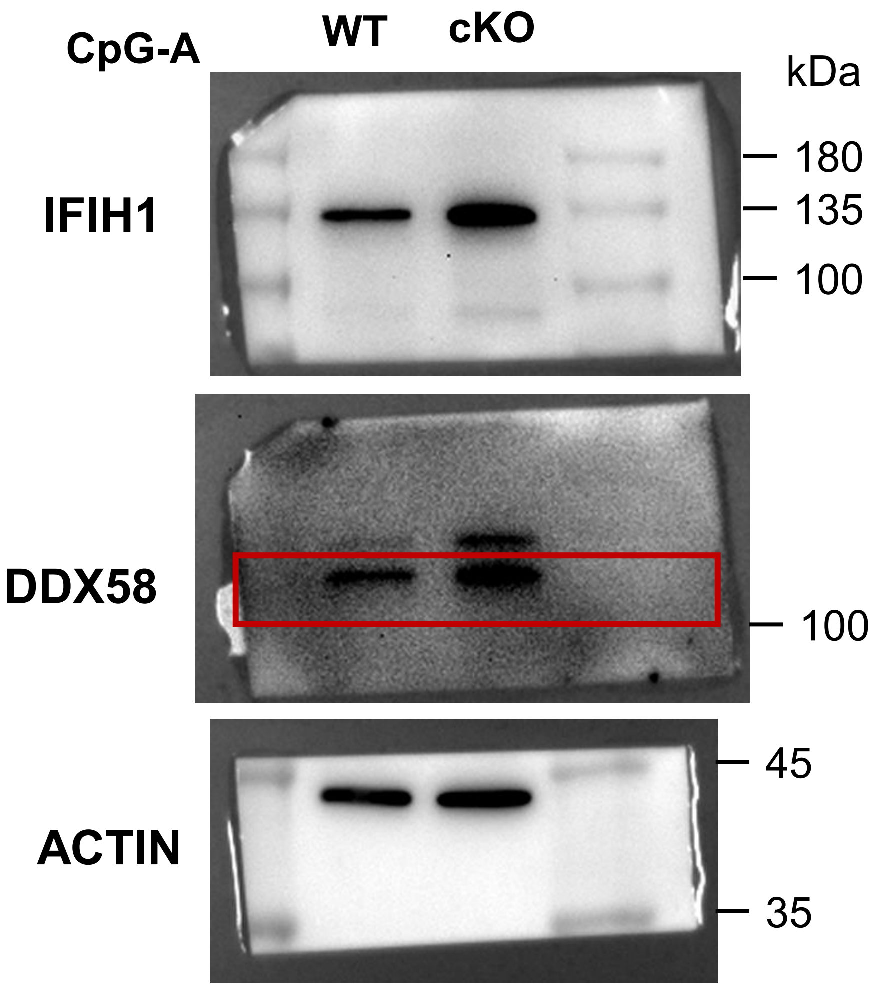

Figure 3. H, I) Western blot analysis of IFIH1 and DDX58 in WT and cKO BM pDCs under the steady state (H) and following 2.5 hours CpG-A (1 μM) stimulation (I).

Source of Figure. 3L

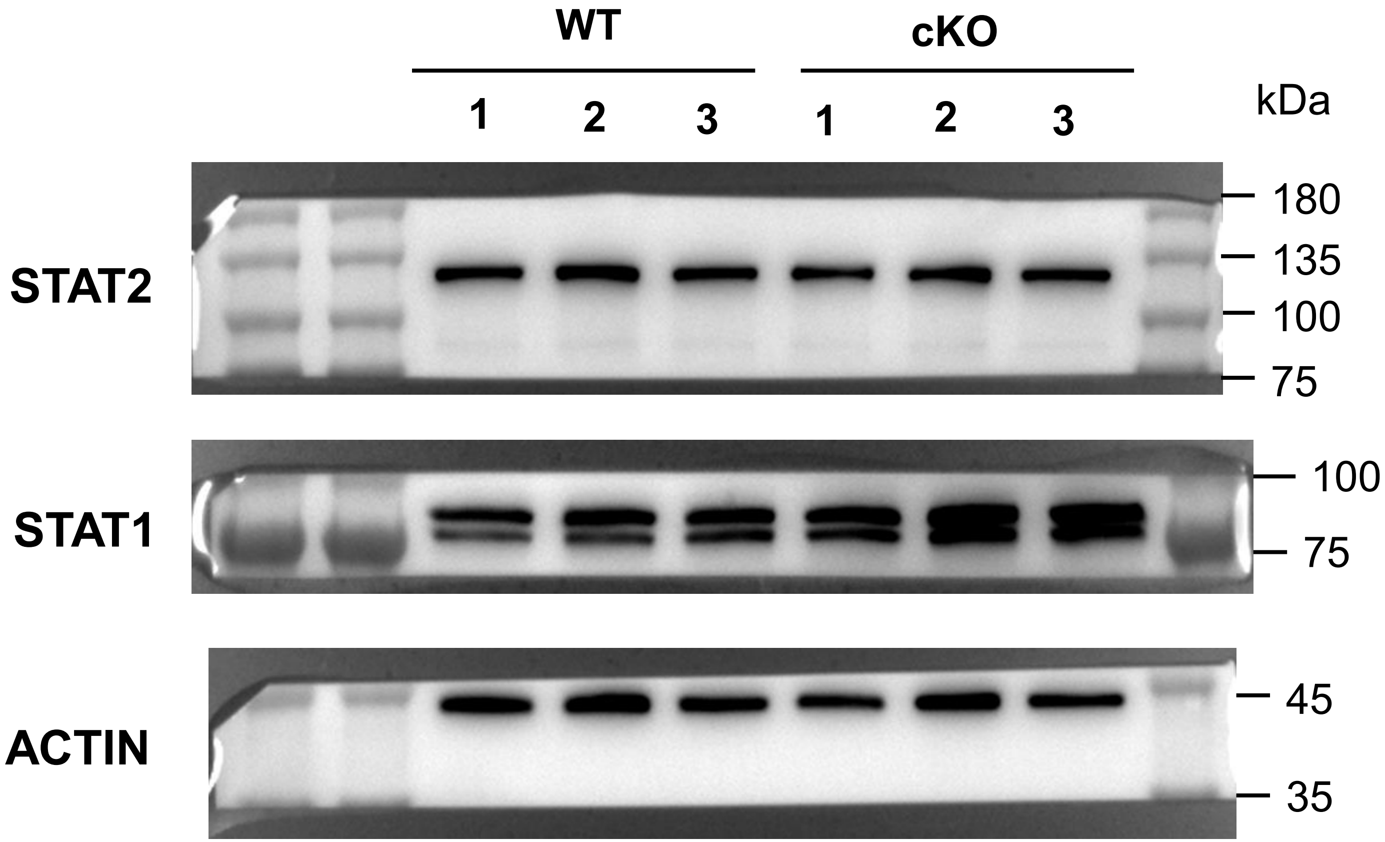

Figure 3. L) Protein levels of STAT1, STAT2 in BM pDCs from WT and cKO mice were analyzed by western blot under steady-state conditions.

Source of Figure. 3M

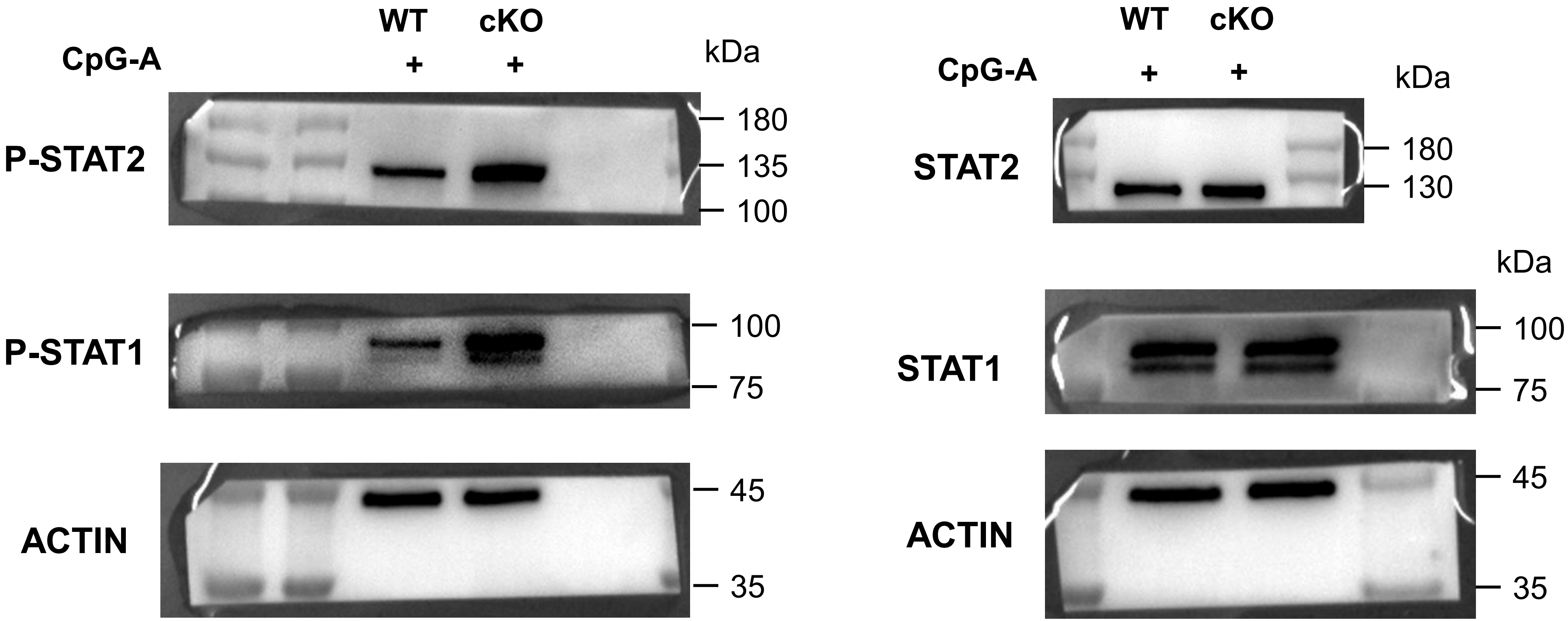

Figure 3. M) Protein levels of STAT1, STAT2, P-STAT1, and P-STAT2 in BM pDCs from WT and cKO mice were analyzed by western blot after 2.5 hours CpG-A (1 μM) stimulation.

Source of Figure. 6F

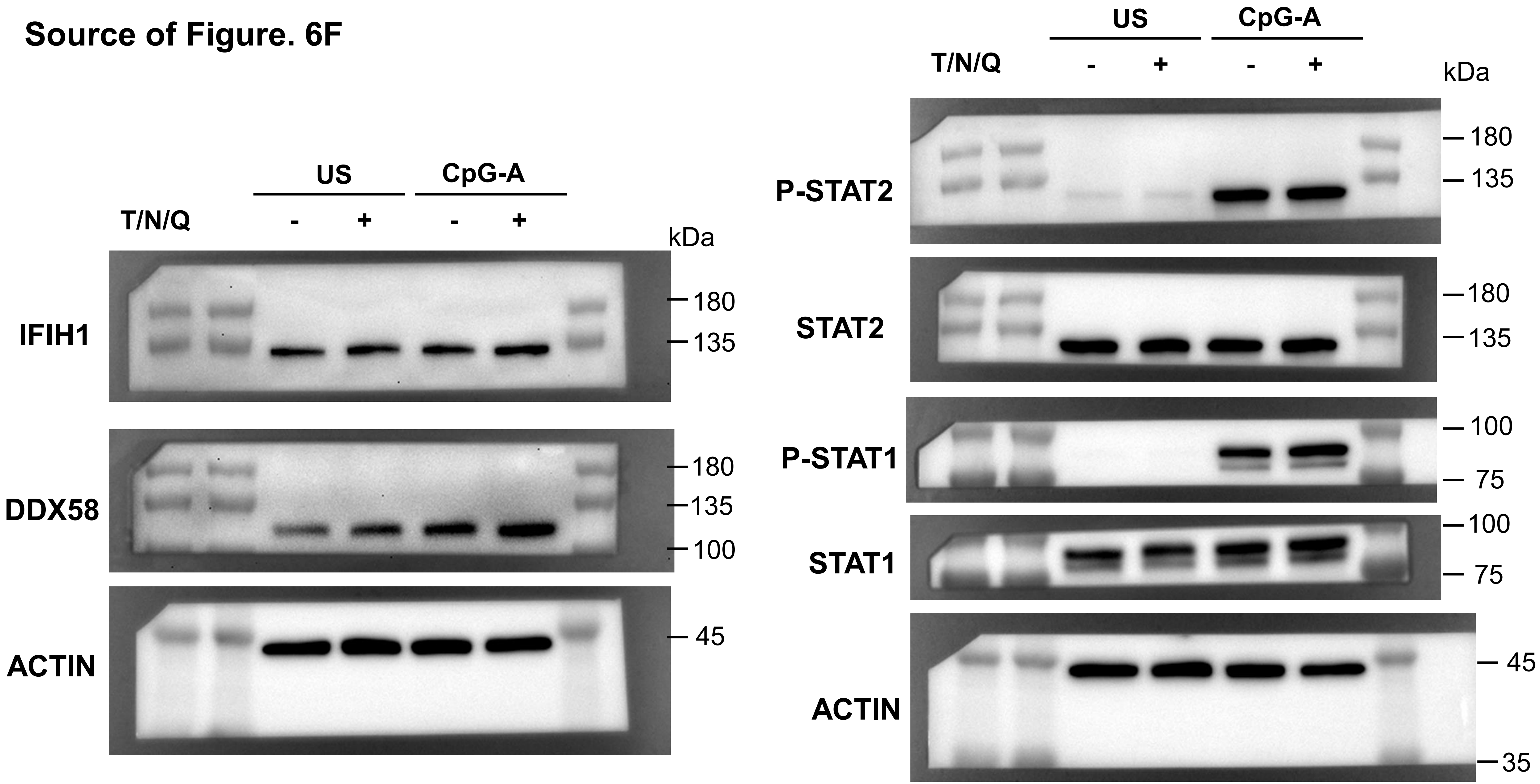

Figure 6. F) Expression of IFIH1, DDX58, STAT1, P-STAT1, STAT2 and P-STAT2 in BM pDCs upon the stimulation of CpG-A (1 μM) with or without three amino acids T/N/Q. US: unstimulated.

Source of Figure S9E

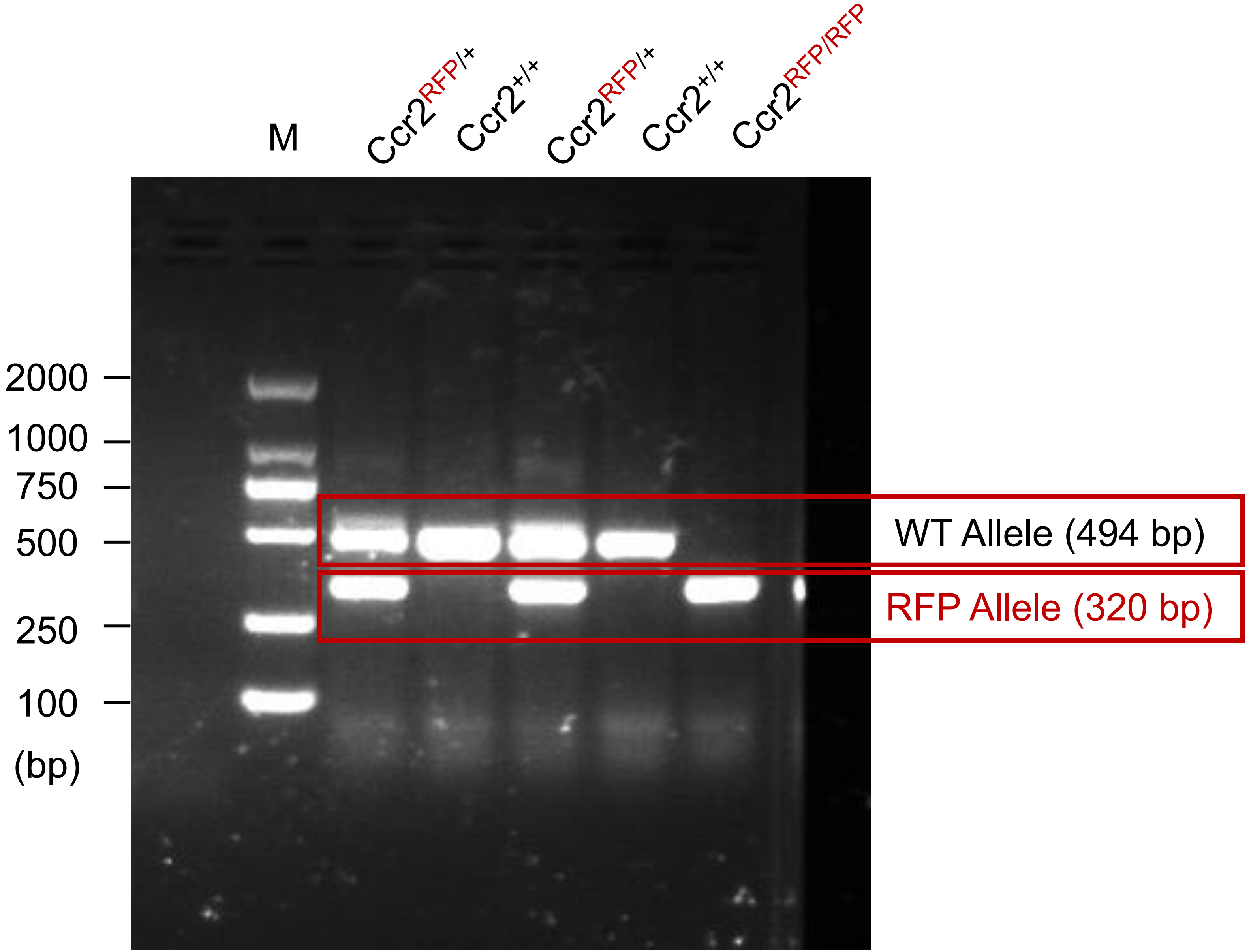

Figure S9. E) Representative genotyping plots for *Ccr2*$^{RFP/RFP}$ knockout mice. The WT allele was 494 bp, and the RFP allele was 320 bp.

Source of Figure S17E

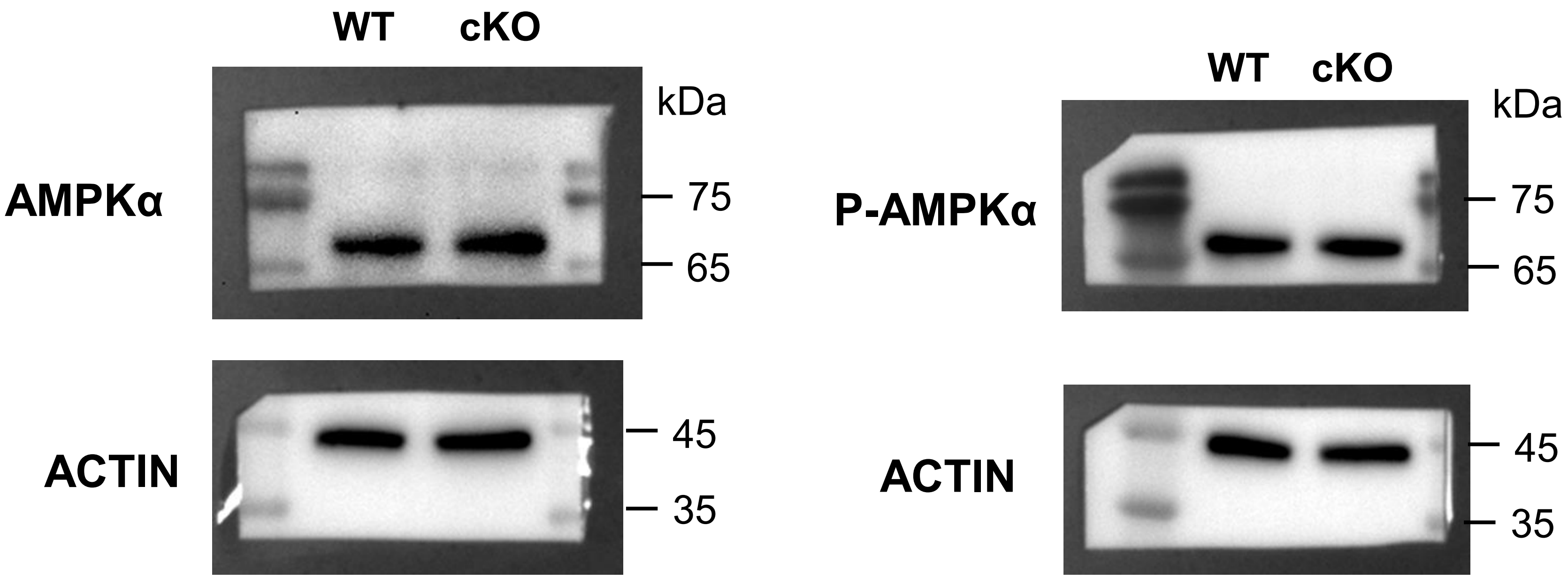

Figure S17. E) Western blot analysis of AMPK and phosphorylated AMPK (P-AMPK) expression in BM pDCs from WT and cKO mice..

Source of Figure S17F

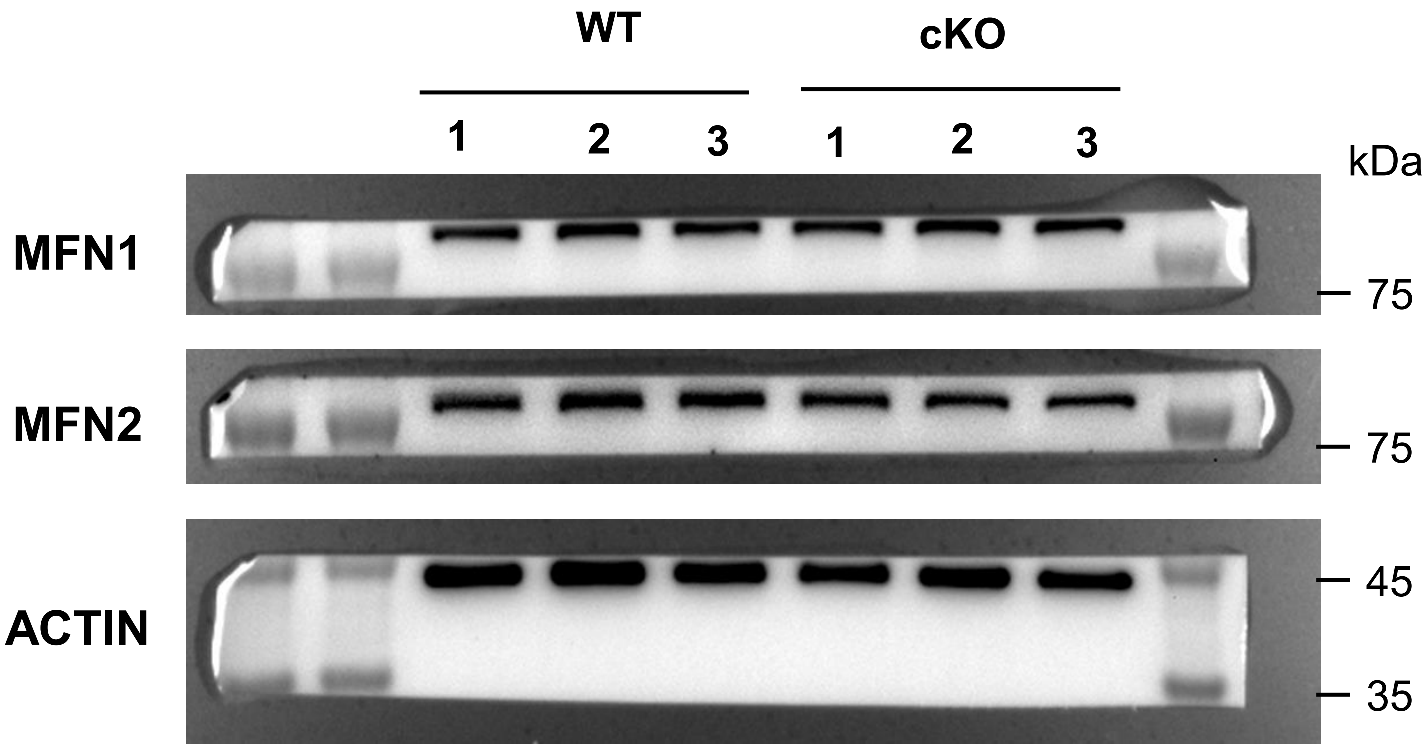

Figure S17. F) The expression of mitochondrial fusion proteins MFN1 and MFN2 in BM pDCs from WT and cKO mice was detected using western blot.

Source of Figure S18A

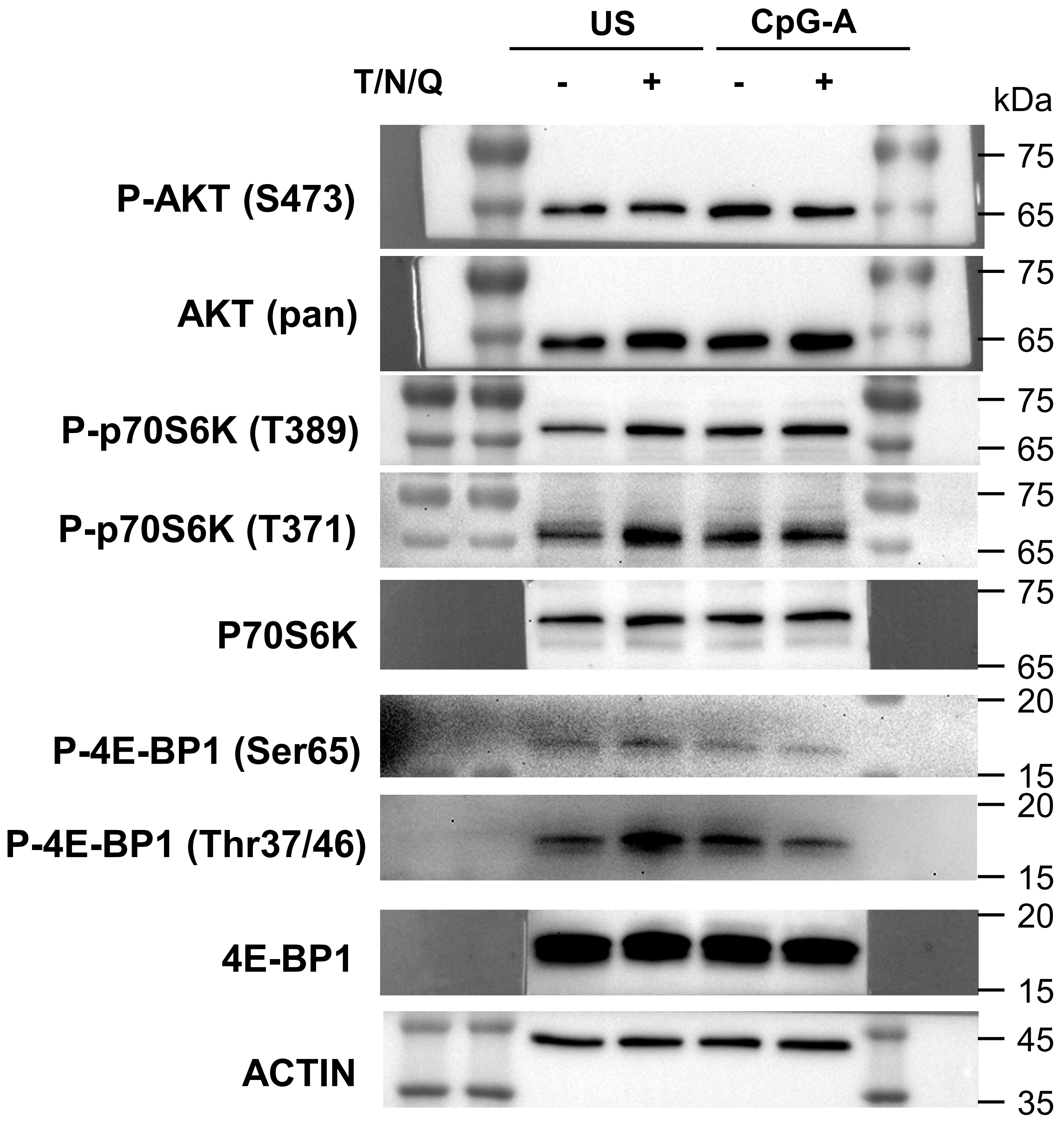

Figure S18. A) Western blot analysis of the expression of P-AKT (S473), AKT, P-p70S6K (T389), P-p70S6K (T371), P70S6K, P-4E-BP1 (Ser65), P-4E-BP1 (Thr37/46), and 4E-BP1 in BM pDCs cultured in complete medium versus T/N/Q-deficient medium following 2.5 hours CpG-A (1 μM) stimulation. US: unstimulated.

Source of Figure S18B

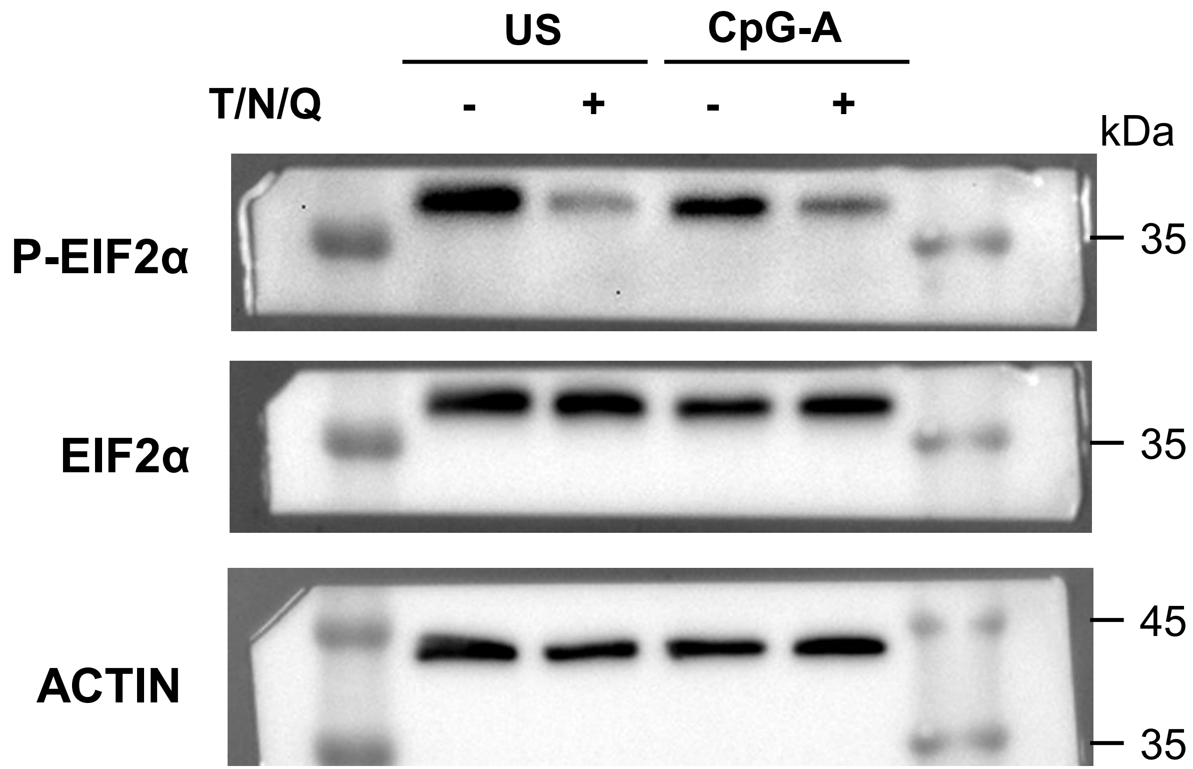

Figure S18. B) Western blot analysis of the expression of EIF2α and phospho-EIF2α (P-EIF2α) in BM pDCs cultured in complete medium versus T/N/Q-deficient medium following 2.5 hours CpG-A (1 μM) stimulation. US: unstimulated.
